# Heterogeneous effects of eccentric training and nordic hamstring exercise on the biceps femoris fascicle length based on ultrasound assessment and extrapolation methods: A systematic review of randomised controlled trials with meta-analyses

**DOI:** 10.1371/journal.pone.0259821

**Published:** 2021-11-09

**Authors:** Gokhan Yagiz, Esedullah Akaras, Hans-Peter Kubis, Julian Andrew Owen

**Affiliations:** 1 School of Human and Behavioural Sciences, Bangor University, Bangor, United Kingdom; 2 Faculty of Health Sciences, Erzurum Technical University, Erzurum, Turkey; 3 Faculty of Health Sciences, Gazi University, Ankara, Turkey; University of Catanzaro: Universita degli Studi Magna Graecia di Catanzaro, ITALY

## Abstract

**Objective:**

To systematically review the effects of eccentric training based on biceps femoris fascicle length using ultrasound assessment and extrapolation methods.

**Design:**

Systematic review and meta-analysis of randomised controlled trials.

**Data sources:**

CENTRAL, CINAHL Plus with full text, PubMed and OpenGrey databases were searched on 6 July 2021.

**Eligibility criteria for selecting studies:**

Randomised controlled trials (RCTs) lasting at least four weeks and presenting data about biceps femoris (BF) fascicle length (FL) as an outcome.

**Method:**

Searching databases, screening studies, performing risk of bias assessments and determining the level of evidence (LoE) for each meta-analysis were applied during the study. PRISMA 2020 statement and Cochrane Handbook for Systematic Reviews of Interventions were used as the guidelines of this systematic review.

**Results:**

Eight randomised controlled trials included in meta-analyses. Based on the very low and low LoE, eccentric training has small (*g* = 0.29, 95% CI [-0.26, 0.85]), moderate (*g* = 0.72, 95% CI [0.17, 1.28]) and large (*g* = 2.20, 95% CI [0.99, 3.41]) effect sizes (ES) based on manual linear extrapolation (MLE), panoramic ultrasound scanning and trigonometric equation methods, respectively. Similarly, Nordic hamstring exercise (NHE) has small (*g* = 0.23 [-1.02, 1.47]), small (*g* = 0.38, 95% CI [-0.50, 1.27]) and large (*g* = 1.98, 95% CI [0.52, 3.44]) ES based on the MLE, panoramic ultrasound scanning and trigonometric equation methods, respectively.

**Conclusion:**

ES of eccentric training, including NHE, vary between the MLE, panoramic ultrasound scanning, and equation methods. The relevant scientific community should have a consensus on measurement standards of the BF FL measurements. Further studies can be conducted to compare the effects of eccentric training based on the ultrasound assessment and extrapolation methods.

## Introduction

Hamstring strain injuries (HSIs) appear as an endemic injury among non-contact injuries for the sports that require high-speed running, including Australian Rules football, rugby union and football [1–5]. Despite increased efforts by researchers to provide an optimal injury prevention technique in the last two decades, HSIs have increased based on earlier epidemiologic data in Australian Rules football, rugby union and football [6]. For instance, Ekstrand and coworkers [7] detected a 4% annual increase in HSIs between 2001 and 2014 in professional football. The biceps femoris long head appears to be the most frequently injured muscle among the hamstring muscles [8]. In addition, re-injuries are very frequent in this anatomical section in the event that an adequate rehabilitation process and an adequate instrumental evaluation have not been performed [9].

The hamstring muscles are important contributors for stabilizing the knee joint, and a more balanced hamstring to quadriceps force ratio is shown to reduce lower limb injury [10–12]. The majority of HSIs occur during running activities [13, 14]. The late swing phase of running was defined as the most vulnerable time for hamstrings [15–17]. During the late swing phase of running, the hamstrings behave as an antagonist to the quadriceps femoris and produce eccentric contraction for controlling quadriceps femoris muscle and for decelerating tibia [18]. At this moment, the biceps femoris is exposed to the highest stretch and reaches about 110% of its length, which is greater than semimembranosus (108.2%) and semitendinosus (107.5) [19]. HSIs generally occur when the muscle fibres cannot resist the excessive tensile force [20]. For this reason, insufficient eccentric contraction of the hamstrings during the late swing phase of running was considered the leading cause of HSIs [15, 21]. In the light of this information, researchers have focused on improving the stated insufficient eccentric contraction of hamstrings and proposed eccentric strength training, including the popular Nordic hamstring exercise (NHE) as an injury prevention strategy for HSIs [22–25]. It should also be noted that there is an ongoing debate about whether hamstrings produces eccentric contraction or isometric contraction during the late swing phase of running [26, 27].

Shorter biceps femoris fascicle length (FL) has recently been proposed as a risk factor for HSIs in 2016 [28]. Timmins et al. [28] highlighted that a biceps femoris FL shorter than 10.56 cm increases the risk of an HSI more than fourfold. Since this date, the number of studies examining the effects of eccentric strength training, including NHE, on the biceps femoris FL has been increasing. Additionally, three systematic reviews and meta-analyses reporting effects of general eccentric strength training on the biceps femoris FL [29] or particularly the effects of the NHE [30, 31] on the biceps femoris FL have been published in the last two years.

In the previous systematic reviews and meta-analyses, Cuthbert et al. [30] claimed that the NHE has a very large effect size of more than 2.58 to increase biceps femoris FL; Medeiros, Marchiori and Baroni [31] reported large effect size (0.97) for the effects of NHE on the same parameter, and Gérard et al. [29] calculated a 1.97 cm eccentric strength training-induced increment in the biceps femoris FL. However, the previous meta-analyses [29–31] pooled the studies without consideration of whether the studies used which ultrasound assessment or extrapolation methods. Furthermore, none of the meta-analyses [29–31] explored the underlying reason for their substantial to considerable statistical heterogeneities [32] (I^2^ = 88.03% [30], I^2^ = 99% [29], I^2^ = 71% [31]) that detected by the I^2^ statistics, which indicates the percentage ratio of the variability in effect estimates caused by heterogeneity rather than chance [32].

Recently, Franchi et al. [33] have compared methods, including panoramic ultrasound scanning (extended field of view (EFOV)), manual linear extrapolation (MLE) and trigonometric equations for estimating biceps femoris FL; they demonstrated that equation methods from a single image significantly overestimate biceps femoris FL compared to the EFOV technique, while no significant difference between EFOV and MLE techniques was observed. Additionally, Franchi et al. [33] criticised the intervention studies used the trigonometric equation method to calculate biceps femoris FL for effects of eccentric training, and reported a high magnitude of biceps femoris FL change.

Despite lacking an intervention study comparing effects of eccentric training on the biceps femoris FL based on estimations via trigonometric equation methods, MLE and panoramic ultrasound scanning, this systematic review aims to recalibrate effect sizes of eccentric training in general and, in particular, effect sizes of the NHE on the biceps femoris FL comparing the ultrasound assessment and extrapolation methods.

## Method

The Preferred Reporting Items for Systematic Reviews and Meta-analyses (PRISMA) 2020 statement was used as the guideline for this study, which is designed on the basis of systematic reviews of randomised controlled trials consisting of a 27-item checklist [34].

### Database search strategy

PubMed, CINAHL Plus with Full Text via Ebsco, The Cochrane Central Register of Controlled Trials (CENTRAL) and OpenGrey databases were searched for all the indicated date range. A combination of the following key terms were used for the database searches: ’Exercis*’, ’Training*’, ’Biceps Femoris’, ’Hamstring*’, ’Knee Flexors’, ’Posterior Thigh’, ’Semitendinosus’, ’Semimembranosus’, ’ACSA’, ’Architectur*’, ’Cross Sectional Area’, ’Cross-sectional Area’, ’Fascic*’, ’Fiber Length’, ’Fibre Length’, ’Pennat*’, ’Pinnat*’, ’Muscle Thickness’, ’Muscle Volume’, ’Muscle Structure’, ’Muscle Length’ and ’PCSA’. When applicable, relevant MeSH terms for ’exercise’ were added to the key terms during the database searches. When "OR" bullion operator was employed within the key term groups, "AND" bullion operator was used between the key term groups. The last search of the databases was conducted on 6 June 2021; all the database searches are shown in the S1 File.

The first author performed the database searches. Once the searches of PubMed, CINAHL Plus with Full Text via Ebsco and The Cochrane Central Register of Controlled Trials (CENTRAL) database were completed, citations were exported to the Endnote^x9^ citation manager [35]. The first author automatically removed duplicate citations through the Endnote citation manager.

### Study selection process and criteria

After removing duplicates, the citations were independently screened based on the title and abstracts by the first and second authors via Rayyan (http://rayyan.qcri.org), a free web and mobile app designed for screening eligible studies for systematic reviews [36]. Additionally, the OpenGrey database was independently screened online on its webpage by the first and second authors. During the study screening period, the first and second authors were blinded to each other’s decisions about all the citations. After screening the studies for eligibility, disagreements regarding selecting eligible studies were resolved by a discussion between the first and second authors. The third and last authors were considered referees for unsolved discussions between the first and second authors for study selection. This process was also applied during the risk of bias assessment and data extraction processes when disagreements arose for selecting eligible studies. Once eligible studies were selected, the lead and second authors also screened reference lists of the included studies.

The following criteria were considered inclusion criteria: (1) being a randomised controlled trial (RCT), (2) eccentric hamstring interventions with at least four weeks of exercise, which was employed by the previous relevant systematic reviews [29–31], (3) presenting effects of eccentric training on biceps femoris FL as an outcome. This systematic review included both sexes as the previous systematic reviews did [29–31], Behan et al. [37] pointed out that biceps femoris FL does not differ between the genders. Additionally, Medeiros, Marchiori and Baroni [31] mentioned that including both sexes is unlikely to impact their meta-analysis.

### Outcome measures

Eccentric exercise-induced alterations in biceps femoris FL based on the ultrasound assessment and extrapolation methods.

### Risk of bias assessments, data extraction and synthesis

The Cochrane Collaboration’s tool for assessing the risk of bias in randomised trials [38] was independently used for determining the risk of bias in included studies by the first and second authors. By following instructions for risk of the bias assessment tool [38], eligible studies were investigated on the basis of random sequence generation (selection bias), allocation concealment (selection bias), blinding participants and personnel (performance bias), blinding outcome assessment (detection bias), incomplete outcome data (attrition bias), selective reporting (reporting bias) and other bias. Each category in this risk of bias assessment tool was graded as ‘low risk of bias,’ ‘unclear risk of bias,’ or ‘high risk of bias’ for each selected study. Afterwards, the decisions were entered into the RevMan computer program [39]. Any conflicts were resolved by the same discussion process for screening eligible studies. Data were independently extracted from included studies by the first and second authors. When a disagreement arose, it was solved through the same discussion mechanism used in the study selection section of this review. The extracted data comprised authors, years, participants’ characteristics, characteristics of exercise interventions, details of ultrasound measurement techniques and results.

Meta-analyses were performed using the Review Manager (RevMan 5.4.1) program [39]. A non-training placebo or control group was considered a comparator for an exercising group in each study. The mean difference (MD) in cm and the standardised mean difference (SMD) in Hedge’s (adjusted) *g* effect size were calculated for each meta-analysis as a summary statistic using RevMan [39]. The SMD used in the review was the effect size, namely, Hedges’ (adjusted) *g* in the RevMan program [40]. Hedges’ *g* differs from Cohen’s d by adjusting effect size and correcting potentially biased estimates in the case of a small sample (n < 20) [41]. The intervention effect size has been interpreted by the following classification: small (0.2), medium (0.5) or large (0.8), which are commonly used for Cohen’s d [42] and Hedges’ g [43] effect size interpretations [44].

The missing standard deviation (SD) is a common feature in studies presenting continuous outcome data [32]. The missing standard deviations of changes from baseline for a group can be calculated using the following formula [32, 45]:

$$SDchange=\sqrt{SD^{2}baseline+SD^{2}final-(2\times r\times SDbaseline\times SDfinal)}$$

SD*change* corresponds to the SD of the mean changes from baseline, SD*baseline* corresponds to the SD of the pre-test, SD*final* represents the SD of the post-test, and the *r* corresponds to the correlations between the SD baseline and SD final measurements; however, this correlation value is not generally presented in studies. Therefore, typically, it is not possible to calculate the SD of changes from baseline based on only having the SD baseline and SD post-intervention values. This systematic review followed the suggestions of the Cochrane Handbook for Systematic Reviews of Interventions from the starting point [32]. First, additional data, e.g., confidence intervals (CI), P values, t values, F values and standard errors, were checked and missing SD changes from baseline were calculated using the Review Manager (RevMan 5.4.1) program when sufficient information was available [39]. However, due to insufficient information, this type of calculation was not possible in most studies in the systematic review. As a second step, the authors of the eligible studies were contacted and asked to share missing relevant data. Before the meta-analyses, FL data of eligible studies was converted into centimetres (cm), to avoid miscalculations of the mean difference changes in meta-analyses.

When a meta-analysis was performed, heterogeneity was assessed by chi-squared (χ^2^, or Chi^2^) statistics. The level of heterogeneity calculated by I^2^ statistics indicates the percentage ratio of the variability in effect estimates caused by heterogeneity rather than chance [32]. 25%, 50%, and 75% I^2^ results were grouped as low, moderate and high, respectively [46]. Meta-analyses were performed using a more conservative random effect (RE) model for continuous data, inverse variance and 95% CI [47]. The random effect model was considered as providing a better account for methodological and statistical heterogeneities in a recent systematic review [48].

After performing meta-analyses, the relevant data were exported to GRADEpro GDT software [49], and the level of a body of evidence (LoE) was assessed by applying the GRADE (Grading of Recommendations, Assessment, Development and Evaluation) approach in the GRADE handbook [50]. The usage of the GRADE approach was recommended by the Cochrane Collaboration’s tool for assessing the risk of bias in randomised trials [38] and the Cochrane Handbook for Systematic Reviews of Interventions [32] for clarifying the level of a body of evidence. The GRADE approach classifies the quality of a body of evidence as high, moderate, low and very low [50]. A GRADE evidence profile was assessed via the GRADEpro GDT software for the levels of the bodies of evidence in consideration of study design, risk of bias, inconsistency, indirectness, imprecision and publication bias.

## Results

### Database search results

**Fig 1:**
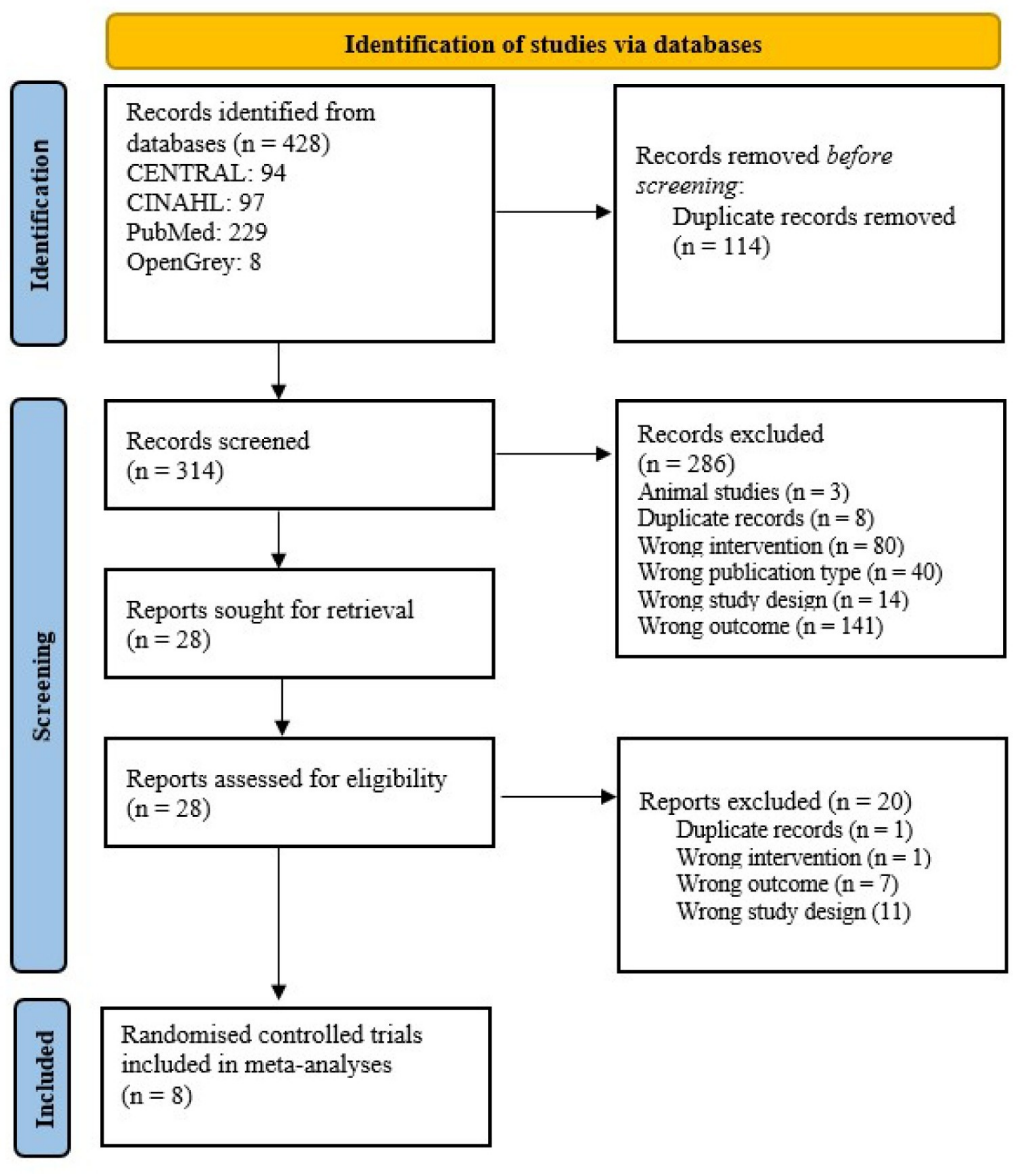
PRISMA 2020 flow diagram. This diagram illustrates the eligible study identification, screening, inclusion and exclusion processes of this systematic review.

Initially, 428 records were identified throughout the database searches. 114 duplicate records were automatically removed via Endnote^x9^ citation manager [35]. The remaining 314 records were screened based on the title and abstracts via the Rayyan web program [36]. Afterwards, 28 records were included in the full-text screening. As a result, eight RCTs [51–58] were included in meta-analyses. The study selection process is illustrated in the PRISMA 2020 flow diagram (Fig 1). Additionally, and a PRISMA 2020 checklist is presented in the S2 File.

### Characteristics of included studies

The study groups, participants’ ages, genders, physical activity levels, training types, total volumes, ultrasound extrapolation techniques, reliability of ultrasound assessments, mean changes and standard deviations of the mean changes between post and pre-tests, and results are presented in Table 1.

**Table 1:** Characteristic of the eligible randomised controlled trials.

RCTs	Groups	Participants’ physical activity level	Mean age	Eccentric training program	Total volume	Extrapolation and ultrasound measurement techniques	Reliability of ultrasound assessments	Post-pre mean (cm)± SD	Significance compared to control group (p-value)
**Bourne et al. [51]**	HE: 10 males	Recreationally active	HE: 23.1± 4.1	HE: 10-weeks of HE exercise	**596 reps (284 for the first 5-weeks)**	**Trigonometric equation**, single image from the mid-thigh	**NA**	**HE**: 1.328± 0.440676 (for the first 5-weeks: 0.75± 0.61857)	HE: **Significant increase at post training** (p = 0.003) **and mid-training** (p = 0.011)
	NHE: 10 males		NHE: 21.6± 3.2	NHE: 10-weeks of NHE			It is stated the assessor has previously shown > 0.90 ICC value elsewhere	**NHE**: 2.218± 0.732132 (for the first 5-weeks: 1.23± 0.461929)	NHE: **Significant increase at post training** (p = 0.001) **and mid-training** (p < 0.001)
	CG: 10 males		CG: 21.3± 3.7						
								**CG**: -0.189± 0.548583 (for the first 5-weeks: -0.27± 0.359833)	
**Lovell et al. [52]**	NHE-BT: 10 males	Amateur football players	23.6± 4.7	12-weeks of NHE	**684 reps**	**Trigonometric equation**, single image from the mid-thigh	**CV: 8.7%**	**NHE-BT**: 1.55034417± 1.1859943	**Not specified**
	NHE-AT: 14 males							**NHE-AT**: -0.627885264987804± 2.11626770438755	
	CG: 10 males								
								**CG**: -0.27138± 2.86589542	
**Marušič et al. [53]**	EG: 18 (12 males, 6 females)	Recreationally active	EG: 24.2 2.1	6- weeks of modified NHE (75⁰ hip flexion) and glider exercise	**128 reps**	**Panoramic ultrasound**	**ICC: 0.92**	**EG:** 0.5722± 0.512	**Significant increase** (p = 0.04)
	CG: 16 (12 males, 4 females)		CG: 23.0 2.8					**CG:** 0.0313± 0.6074	
**Mendiguchia et al. [54]**	NHE: 7 (gender is not specified)	Football players		6-weeks of NHE	**358 reps**	**Manual linear extrapolation**, single image from the mid-thigh	**ICC:** 0.989	**EG:** 0.73 ± 1.04882656	**Not specified**
	CG: 8 (gender is not specified)							**CG:** -0.03 ± 0.4670603	
**Potier et al. [55]**	EG: 11 (7 females, 4 males)	NA	EG: 27± 0.8	8-weeks of eccentric hamstring curls	**NA**	**Manual linear extrapolation**, single image, the exact location is not specified	**ICC: 0.95**	**EG:** 1.98± 1.1639	**No significant change** (p = 0.11)
	CG: 11 (9 females, 2 males)		CG: 29.6± 1.2					**CG:** 0.95± 1.6788	
**Riberio-Alvares et al. [56]**	NHE: 10 (7 females, 3 males)	Physically active	NHE: 23.7± 3.3	4-weeks of NHE	**93 reps**	**trigonometric equation,** single image from the mid-thigh	**NA**	NHE: 1.8± 0.93	**Not specified**
	CG: 10 (7 females, 3 males)		CG: 26± 2.7					CG: 0.19± 0.68	
**Seymore et al. [57]**	NHE: 10 (6 females, 4 males)	Recreationally active	NHE: 18.3 ± 0.5	6-weeks of NHE	**358 reps**	**Panoramic ultrasound**	**ICC: 0.99**	NHE: 0.11± 0.9	**No significant change** (p = 0.377)
								CG: -0.18± 0.49	
	CG: 10 (8 females 2 males)		CG: 19.9 ± 1.2						
**Wiesinger et al. [58]**	Eccentric IK: 10 Males	Recreationally active	Eccentric IK: 25.9± 2.6	Eccentric IK: 6-weeks of eccentric exercise at an isokinetic machine	**220 reps**	**Manual linear extrapolation**, single image from the mid-thigh	**NA**	**Eccentric IK:** 0.05± 0.07	Eccentric IK: **No significant change**
	NHE: 10 males		NHE: 25.0± 2.9					**NHE:** -0.01± 0.13	NHE: **No significant change**
	CG: 10 males		CG: 26.2± 2.3	NHE: 6-weeks of NHE				**CG:** 0.04± 0.13	(for overall group x time interaction, p = 0.451)

Note: The mean changes and standard deviations of the mean changes presented in the table were obtained via contacting corresponding authors of the studies Bourne et al. [51], Lovell et al. [52], Marušič et al. [53], Riberio-Alvares et al. [56] and Seymore et al. [57] due to the missing standard deviations of the mean changes. The data presented for the study of Potier et al. [55] was able to be calculated based on the given in-text details via RevMan 5.4.1 [39]. There was no missing outcome data in the publications of Wiesinger et al. [58] and Mendiguchia et al. [54].

Abbreviations: CG, Control group, CV, Coefficient of variations, EG, Exercise group, ICC, Interclass correlation coefficient, IK, Isokinetic, HE, Hip extension, NA, Not applicable, NHE, Nordic hamstring exercise, NHE-AT, Nordic hamstring exercise after training, NHE-BT, Nordic hamstring exercise before training, RCTs: Randomised controlled trials, reps, repetitions.

### Risk of bias assessments

**Fig 2:**
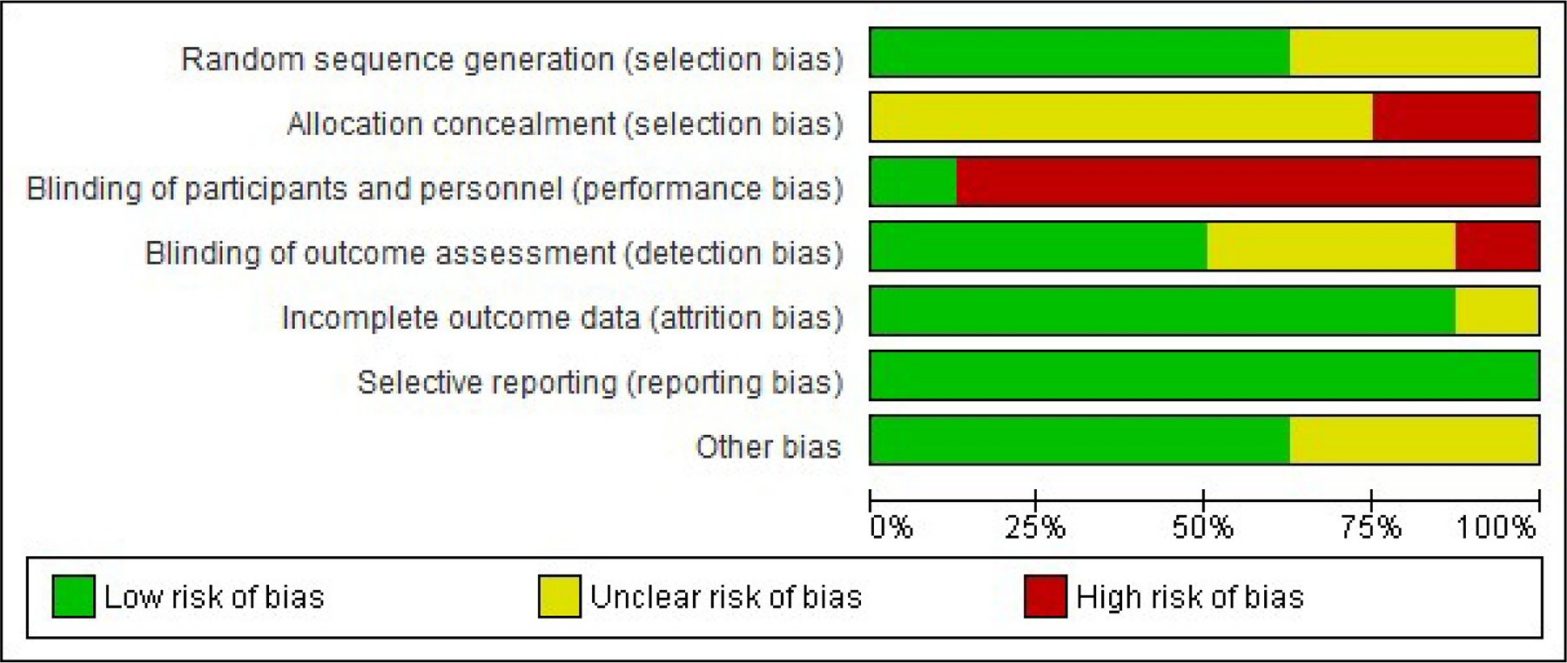
Risk of bias assessment graph. This graph shows the general percentage ratio of reviewer authors’ judgements about the risk of bias of each bias item for all included studies (generated via RevMan 5.4.1).

**Fig 3:**
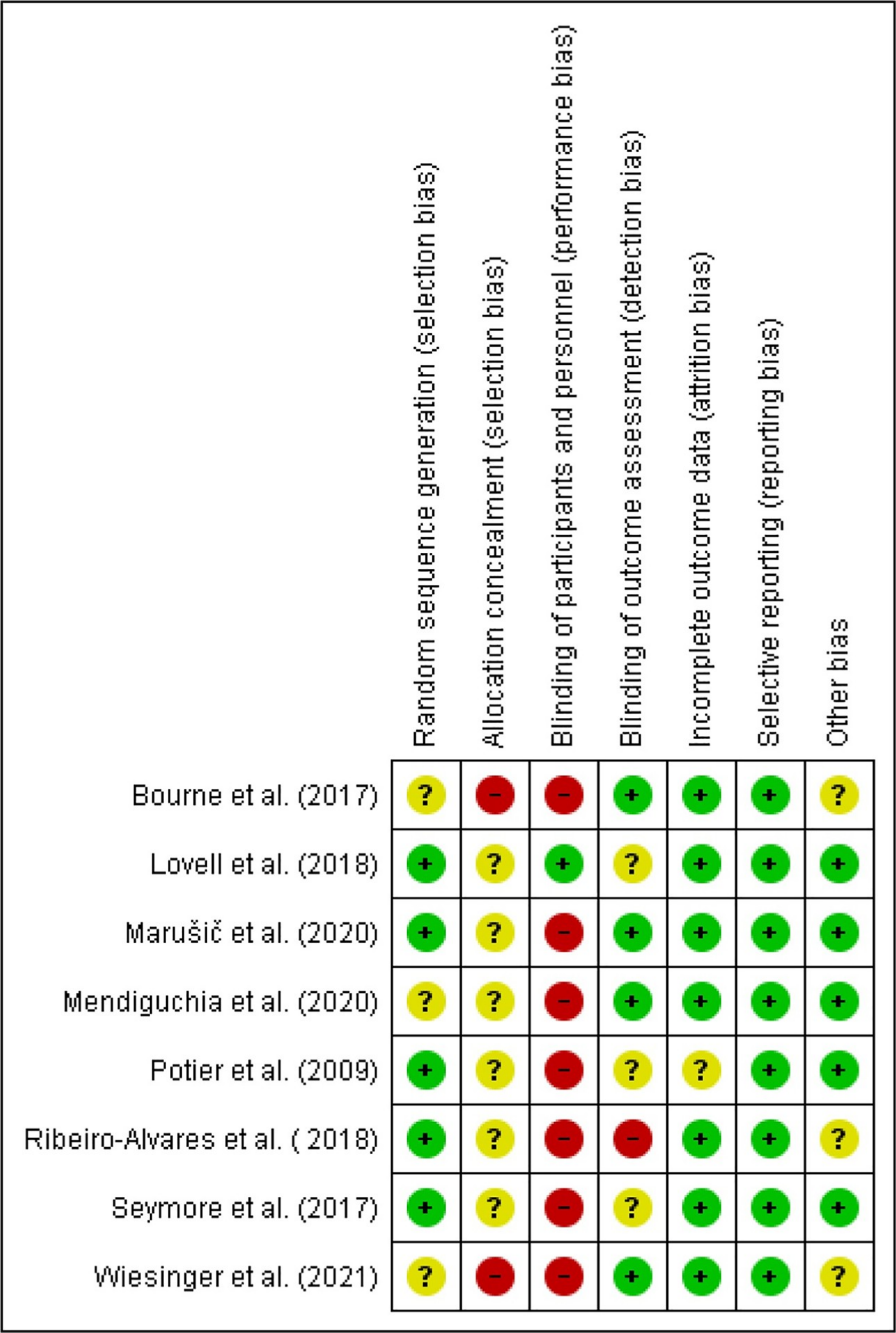
Review authors’ judgements about each risk of bias item for included studies [51–58]. Positive (+) values represent a low risk of bias, question marks (?) represent an unclear risk of bias, and negative (-) values represent a high risk of bias (generated via RevMan 5.4.1).

The first and second authors independently completed risk of bias assessments for each included study via the Cochrane Collaboration’s tool for assessing the risk of bias in randomised trials [38]. The low risk of bias scores of the studies in the seven sections [38] ranged from three [51, 55, 56, 58] to five [52, 53]. The risk of bias assessment graph (Fig 2) and a table showing the authors’ conclusions on the each risk of bias parameter for each study (Fig 3) were generated via RevMan [39] for future use to determine the level of evidence for meta-analyses via GRADEpro GDT software [49].

### Evidence levels of the meta-analyses

The LoE of meta-analyses was determined using the GRADEpro GDT software based on the GRADE approach [50], which categorised the level of a body of evidence as high, moderate, low and very low [50]. The results for each meta-analysis are presented in S3 File.

### Meta-analyses

In total, eight RCTs [51–58] were included in the meta-analyses for effects of eccentric training on biceps femoris FL, and six RCTs [51, 52, 54, 56–58] were included in the meta-analyses for effects of the NHE on biceps femoris FL. Concerning the study of Lovell et al. [52], the FL values of the after training-NHE group were not included in meta-analyses for maintaining methodological homogeneity among the studies. The other pooled studies [51, 54, 56–58] in the meta-analyses investigating the effects of Nordic hamstring exercise on the biceps femoris muscle architecture did not perform the NHE after a sports training. In support, the FIFA 11+ program has prescribed the Nordic hamstring exercise before training [59].

### Effects of the eccentric training based on the ultrasound assessment and extrapolation methods

**Fig 4:**
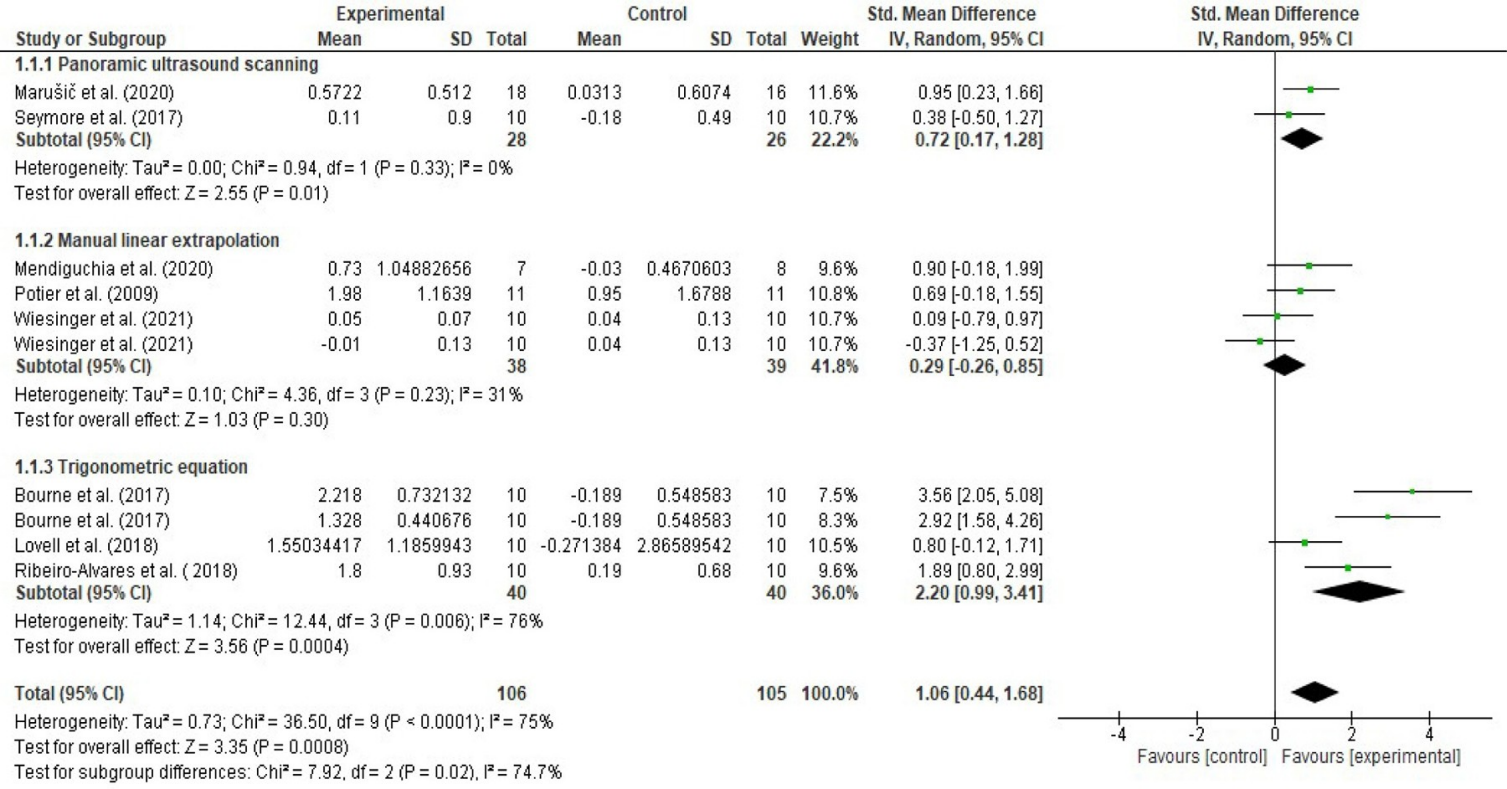
Forest plot effect sizes of eccentric training on biceps femoris fascicle length based on ultrasound assessment and extrapolation methods. Overall eccentric training has a large effect size on increasing biceps femoris FL (*g* = 1.06 [0.44, 1.68], I^2^ = 75%). Eccentric training has a small effect based on the manual linear extrapolation method (*g* = 0.29 [-0.26, 0.85], I^2^ = 31%), a medium effect based on the panoramic ultrasound assessments (*g* = 0.72 [0.17, 1.28], I^2^ = 0%) and a large effect based on the trigonometric equation method (*g* = 2.20 [0.99, 3.41], I^2^ = 76%) (created via RevMan 5.4.1).

**Fig 5:**
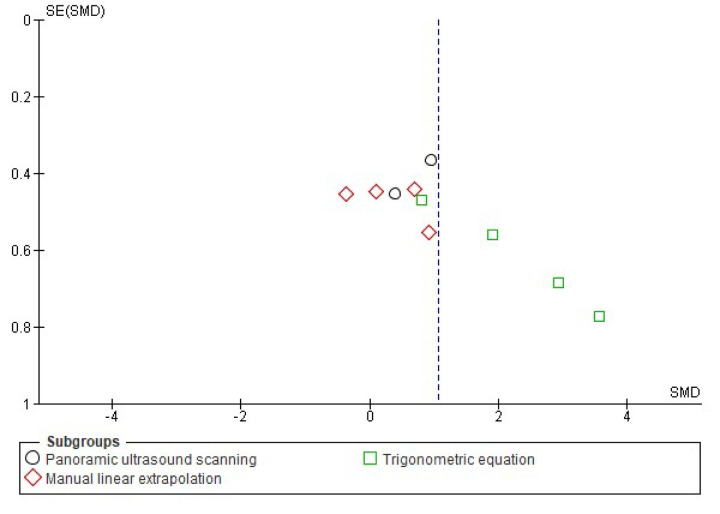
Funnel plot effect sizes of eccentric training on biceps femoris fascicle length based on the ultrasound assessment and extrapolation methods. Red coloured squares represent studies that used manual linear extrapolation method, black coloured circles represent studies that used panoramic ultrasound scanning method, and green coloured squares represent studies that used trigonometric equation method. The asymmetry in the figure means a publication bias between the study groups that were used different ultrasound assessment methods. (created via RevMan 5.4.1). Acronyms: SE(SMD), standard error of standardised mean differences; SMD, standardised mean difference.

**Fig 6:**
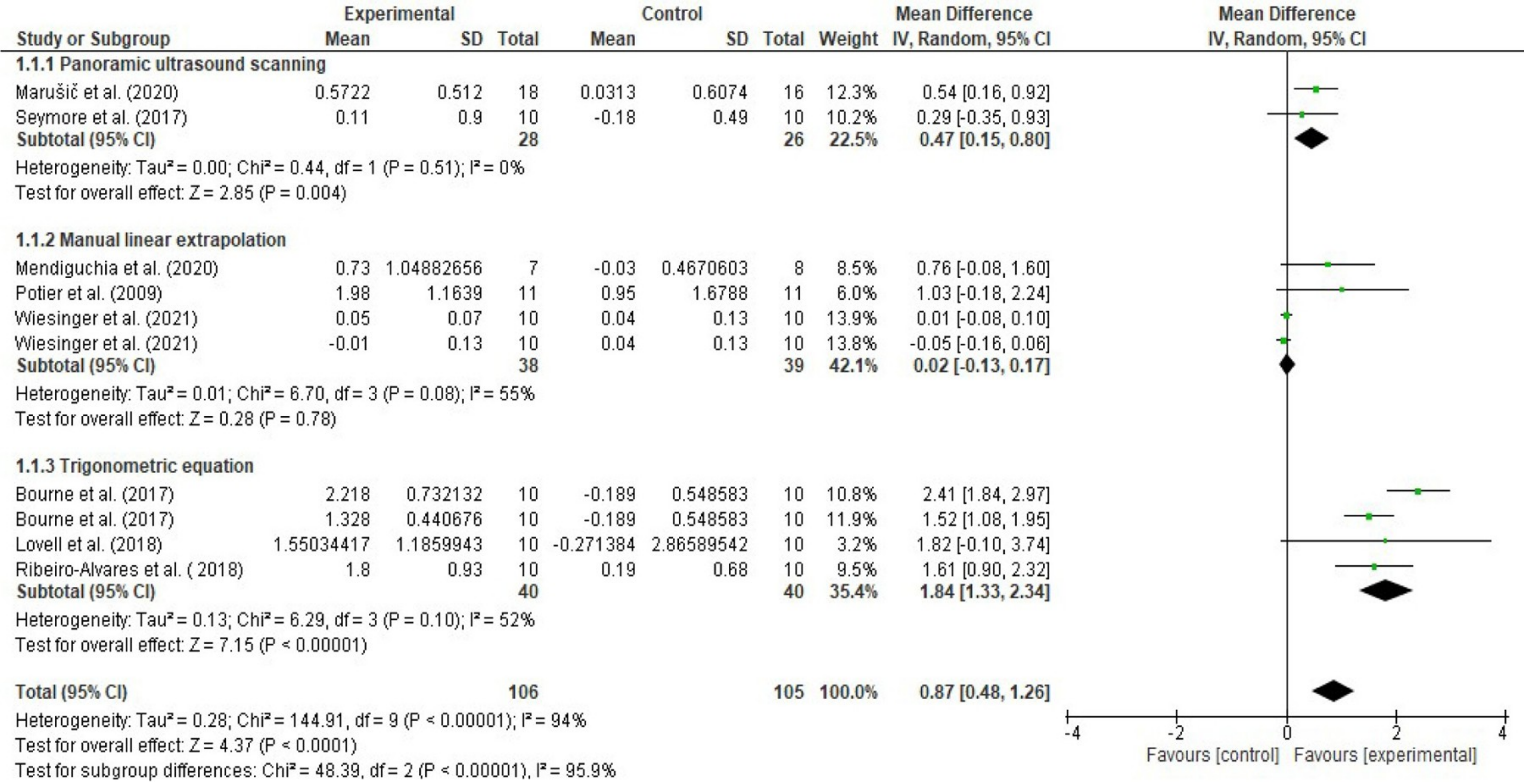
Forest plot eccentric training-induced mean (cm) changes in biceps femoris fascicle length based on the ultrasound assessment and extrapolation methods. Eccentric training leads 0.02 cm ([-0.13, 0.17], I^2^ = 55%), 0.47 cm ([0.15, 0.80], I^2^ = 0%), and 1.84 cm ([1.33, 2.34], I^2^ = 52%) increases in biceps femoris FL based on the MLE method, panoramic ultrasound scanning and trigonometric equation methods, respectively (created via RevMan 5.4.1).

**Fig 7:**
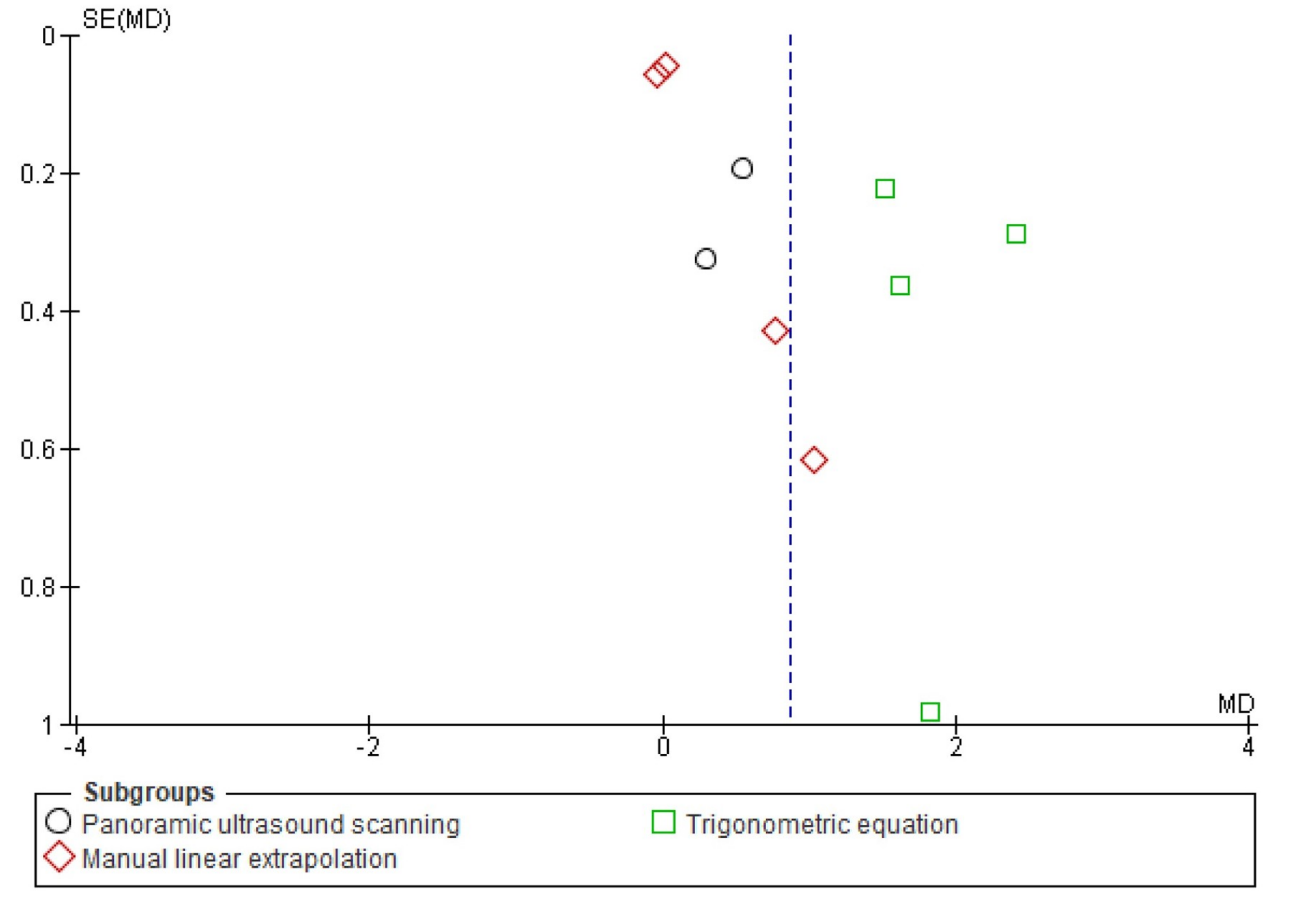
Funnel plot eccentric training-induced mean (cm) changes in biceps femoris fascicle length based on the ultrasound assessment and extrapolation methods. Red coloured squares represent studies that used manual linear extrapolation method, black coloured circles represent studies that used panoramic ultrasound scanning method, and green coloured squares represent studies that used trigonometric equation method. The asymmetry in the figure means a publication bias between the study groups that were used different ultrasound assessment methods (created via RevMan 5.4.1). Acronyms: SE(MD), standard error of mean differences; MD, mean difference.

Eight RCTs [51–58] were included in the meta-analysis assessing the effects of eccentric training on the biceps femoris FL. In future subgroup analyses, three [51, 52, 56] of the RCTs were included in the trigonometric equation subgroup. Three RCTs [54, 55, 58] were included in the manual linear extrapolation (MLE) subgroup, and the remaining two RCTs [53, 57] were included in the panoramic ultrasound scanning subgroup. Hedge’s (adjusted) g effect sizes were calculated for the random effect model and 95% CI for overall effects of eccentric training, effects of eccentric training based on ultrasound equation, linear extrapolation and panoramic ultrasound assessment methods (Figs 4 and 5). Additionally, mean (cm) changes in biceps femoris FL for overall eccentric training and for the same subgroups were calculated and presented in Figs 6 and 7.

Meta-analyses revealed that overall eccentric training has a large effect size on increasing biceps femoris FL (*g* = 1.06 [0.44, 1.68], I^2^ = 75%, LoE = very low). However, subgroup analyses suggested that the effect size of eccentric training on the biceps femoris FL differs from each other based on the ultrasound assessment and extrapolation methods (I^2^ = 74.7%) (Fig 4), ranging from small to large based on the ultrasound assessment and extrapolation methods for assessing biceps femoris FL (Fig 4). Meta-analyses results showed that eccentric training has a small effect based on the MLE method (*g* = 0.29 [-0.26, 0.85], I^2^ = 31%, LoE = low), a medium effect based on the panoramic ultrasound assessments (*g* = 0.72 [0.17, 1.28]), I^2^ = 0%, LoE = low) and a large effect based on the trigonometric equation method (*g* = 2.20 [0.99, 3.41], I^2^ = 76%, LoE = very low) (Fig 4).

Likewise, meta-analyses that were carried out to assess eccentric training-induced MDs (cm) detected differences in the eccentric training-induced cm changes in biceps femoris FL between the ultrasound assessments and extrapolations (I^2^ = 95.9) (Fig 6). Subgroup analyses indicated that eccentric training leads 0.02 cm ([-0.13, 0.17], I^2^ = 55%), 0.47 cm ([0.15, 0.80], I^2^ = 0%), and 1.84 cm ([1.33, 2.34], I^2^ = 52%) increases in biceps femoris FL based on the MLE method, panoramic ultrasound scanning and trigonometric equation methods, respectively (Figs 6 and 7).

### Effects of the NHE based on the ultrasound assessment and extrapolation methods

**Fig 8:**
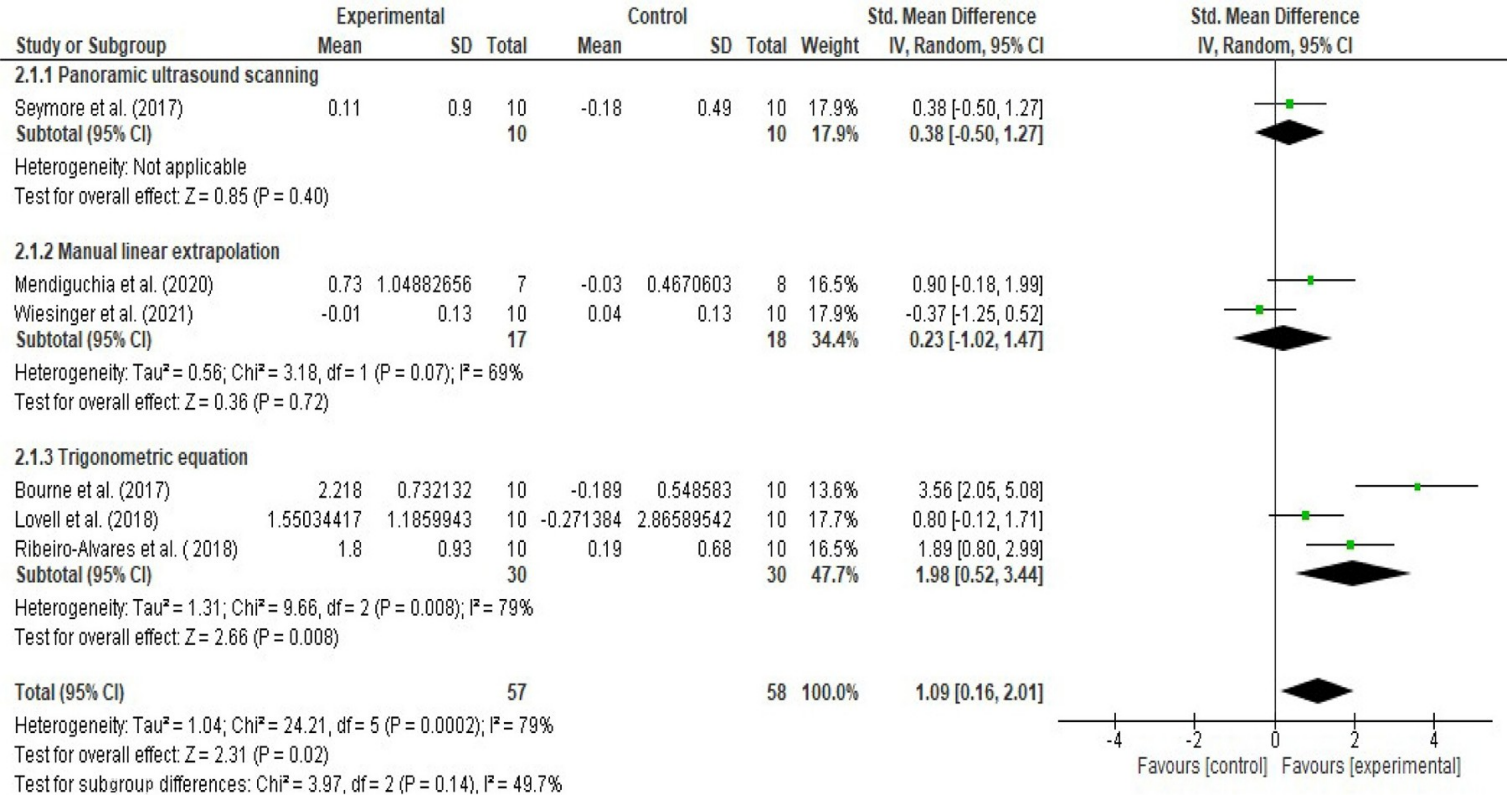
Forest plot effect sizes Nordic Hamstring Exercise (NHE) on biceps femoris fascicle length based on the ultrasound assessment and extrapolation methods. The overall effect size of the NHE on increasing biceps femoris FL was large (*g* = 1.09 [0.16, 2.01], I2 = 79%). NHE has a small effect size on increasing the biceps femoris FL based on the MLE method (*g* =, 0.23 [-1.02, 1.47], I^2^ = 69%), has a small effect size on increasing biceps femoris FL based on the panoramic ultrasound scanning (g = 0.38 [-0.50, 1.27]), and has a large effect on increasing biceps femoris FL based on the equation methods (g = 1.98 [0.52, 3.44], I^2^ = 79%) (created via RevMan 5.4.1).

**Fig 9:**
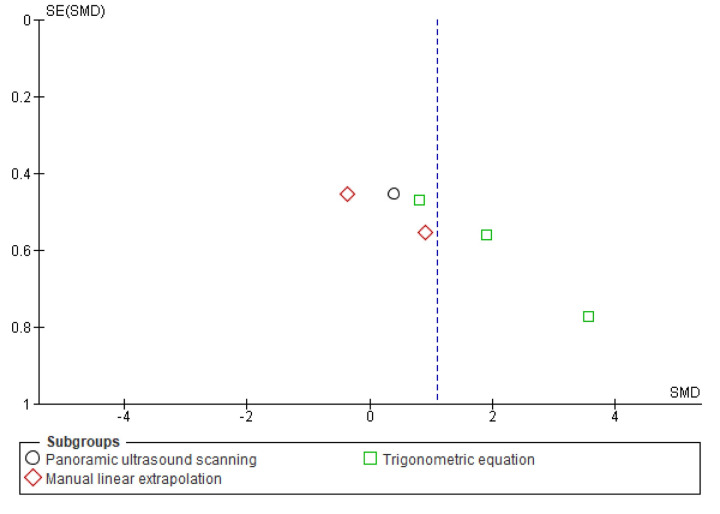
Funnel plot effect sizes Nordic hamstring exercise on biceps femoris fascicle length based on the ultrasound assessment and extrapolation methods. Red coloured squares represent studies that used manual linear extrapolation method, black coloured circles represent studies that used panoramic ultrasound scanning method, and green coloured squares represent studies that used trigonometric equation method. The asymmetry in the figure means a publication bias between the study groups that were used different ultrasound assessment methods (created via RevMan 5.4.1). Acronyms: SE(SMD), standard error of standardised mean differences; SMD, standardised mean difference.

Six RCTs [51, 52, 54, 56–58] were included in the meta-analyses that examine the effects of NHE on the biceps femoris FL. A subgroup analysis was performed for the same parameters of the meta-analyses for eccentric training. The overall effect size of the NHE on increasing biceps femoris FL was large (*g* = 1.09 [0.16, 2.01], I2 = 79%, LoE = very low) (Fig 8). However, the subgroup analysis suggests a difference between the values of the ultrasound assessment and extrapolation methods (Figs 8 and 9). In particular, NHE has a small effect size on increasing the biceps femoris FL based on the MLE method (*g* =, 0.23 [-1.02, 1.47], I^2^ = 69%, LoE = very low), has a small effect size on increasing biceps femoris FL based on the panoramic ultrasound scanning (g = 0.38 [-0.50, 1.27], LoE = low), and has a large effect on increasing biceps femoris FL based on the equation methods (g = 1.98 [0.52, 3.44], I^2^ = 79%, LoE = very low) (Fig 8).

**Fig 10:**
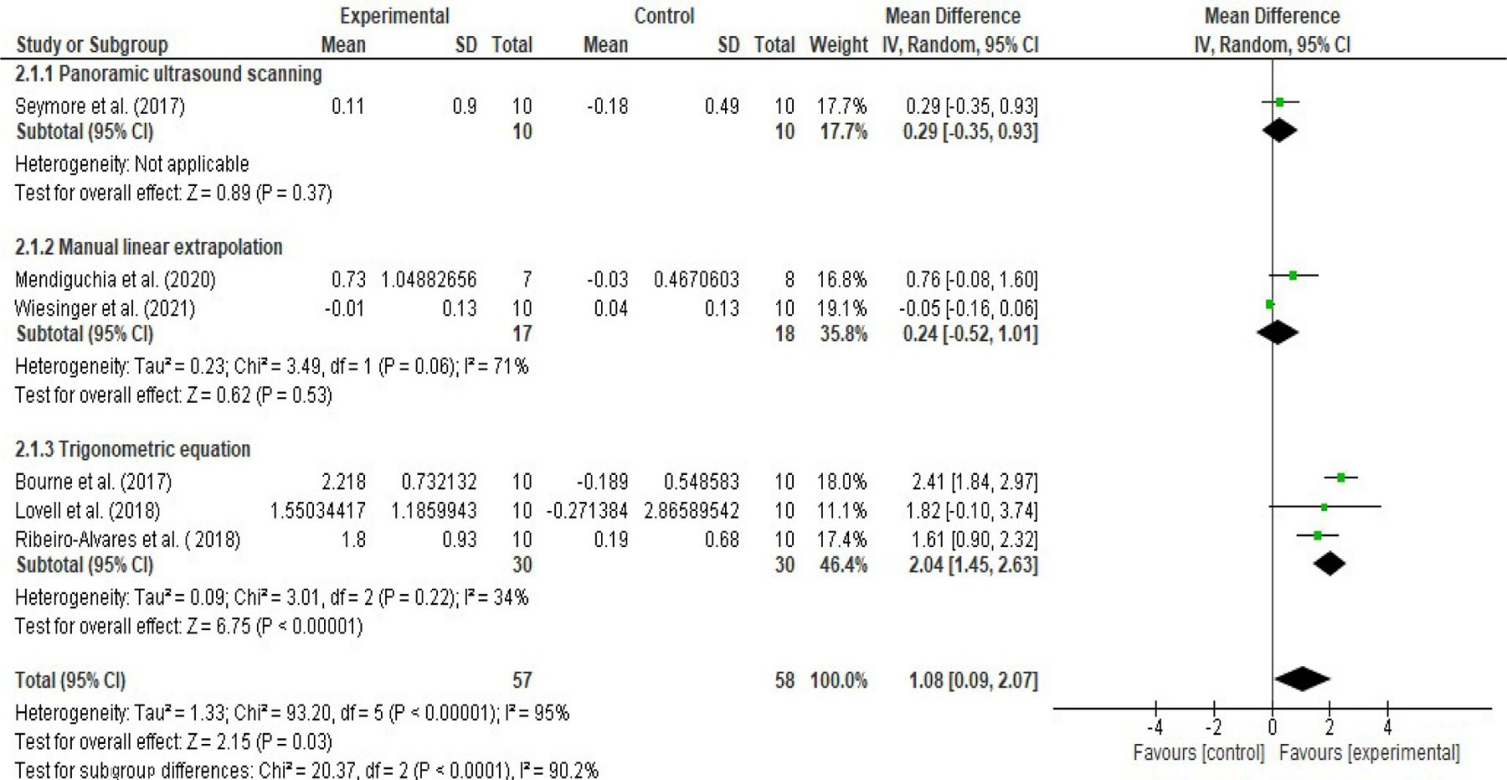
Forest plot Nordic hamstring exercise-induced mean (cm) changes in biceps femoris fascicle length based on the ultrasound assessment and extrapolation methods. Overall, NHE leads to 1.08 cm increment ([0.09, 2.07], I^2^ = 95%). NHE do leads to 0.24 cm ([-0.52, 1.01], I^2^ = 71%), 0.29 cm ([-0.35, 0.93]) and 2.04 cm ([1.45, 2.63], I^2^ = 34%) increases in the biceps femoris fascicle length based on the MLE, panoramic ultrasound scanning and trigonometric equation methods, respectively (created via RevMan 5.4.1).

**Fig 11:**
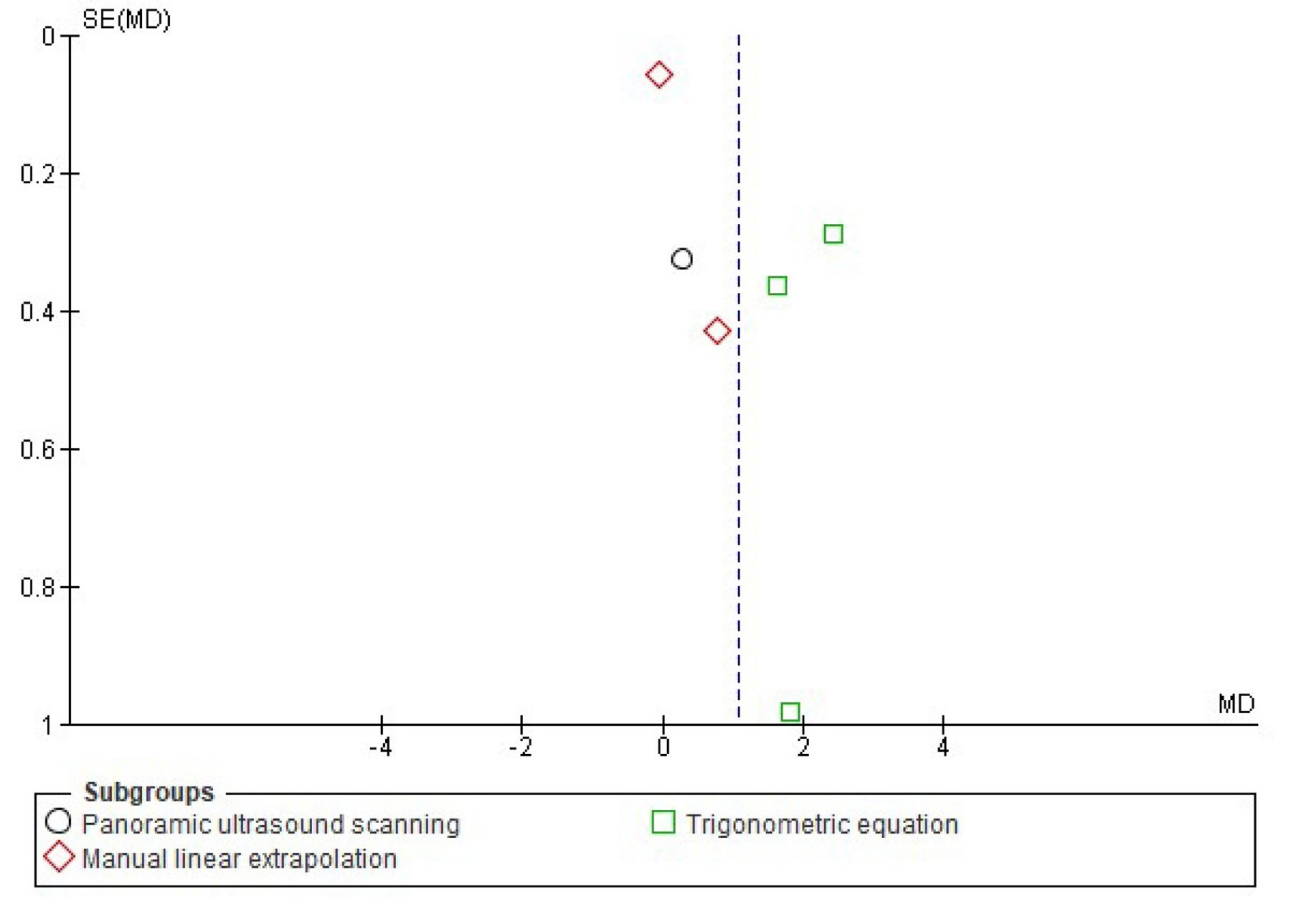
Funnel plot Nordic hamstring exercise induced mean (cm) changes in biceps femoris fascicle length based on the ultrasound assessment and extrapolation methods. Red coloured squares represent studies that used manual linear extrapolation method, black coloured circles represent studies that used panoramic ultrasound scanning method, and green coloured squares represent studies that used trigonometric equation method. The asymmetry in the figure means a publication bias between the study groups that were used different ultrasound assessment methods (created via RevMan 5.4.1). Acronyms: SE(MD), standard error of mean differences; MD, mean difference.

Moreover, the meta-analyses performed to detect the NHE-induced mean (cm) changes found that the NHE leads to 1.08 cm increment ([0.09, 2.07], I^2^ = 95%) in the biceps femoris FL (Figs 10 and 11). However, subgroup analysis indicated considerable differences between the study groups applied equation, MLE and panoramic ultrasound techniques (I^2^ = 90.2%) (Fig 10). Subgroup analysis showed that the NHE do leads to 0.24 cm ([-0.52, 1.01], I^2^ = 71%), 0.29 cm ([-0.35, 0.93]) and 2.04 cm ([1.45, 2.63], I^2^ = 34%) increases in the biceps femoris fascicle length based on the MLE, panoramic ultrasound scanning and trigonometric equation methods, respectively (Fig 10).

### Effects of 4–6 weeks of NHE on the biceps femoris FL based on ultrasound assessment and extrapolation methods

**Fig 12:**
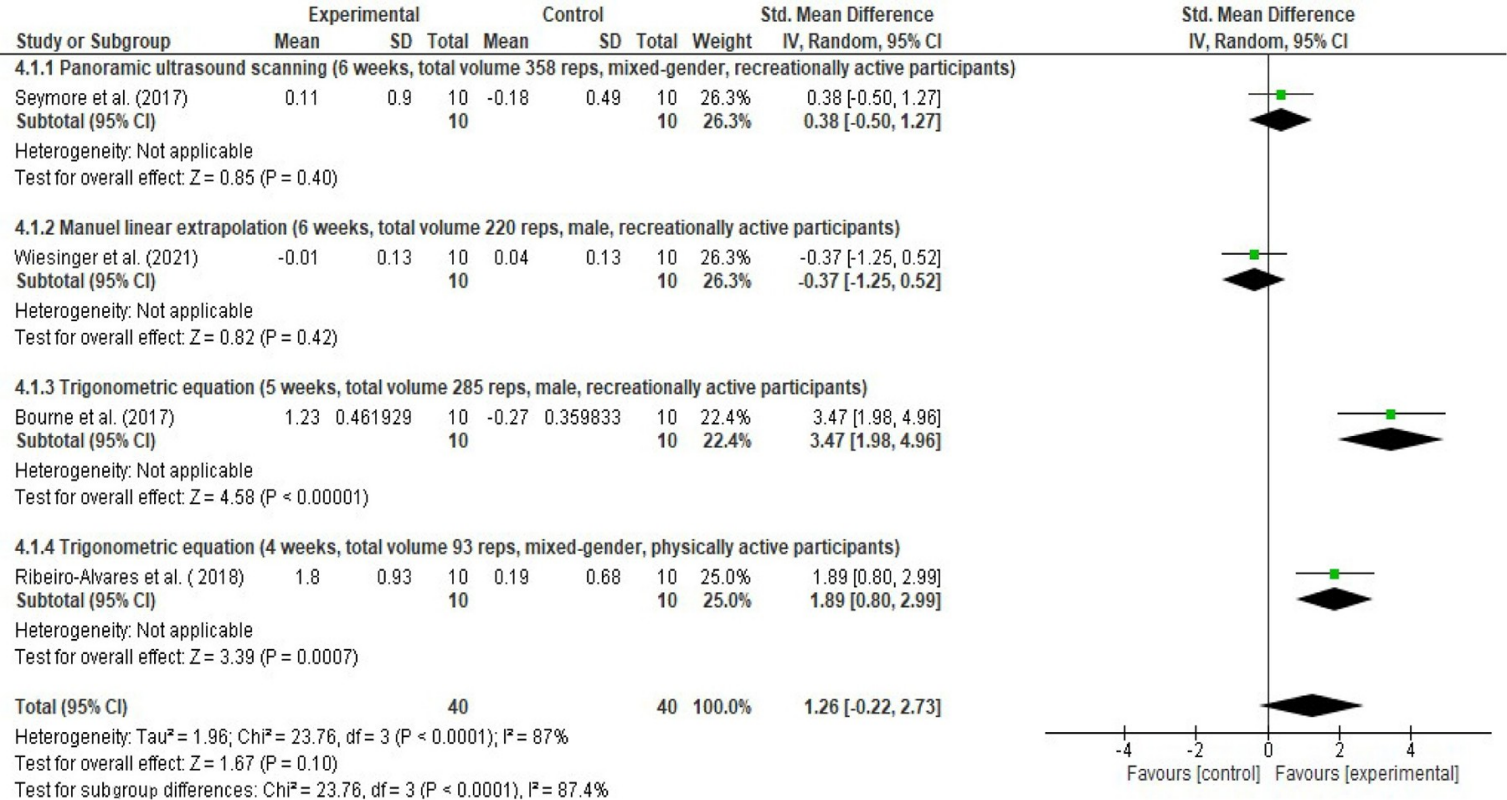
Forest plot effects of 4–6 weeks of Nordic hamstring exercise on the biceps femoris FL based on ultrasound assessment and extrapolation methods. NHE interventions used trigonometric equation methods for estimating the FL showed large effects sizes (*g* = 1.89–3.47) on increasing biceps femoris FL, while the six weeks of NHE interventions using the MLE (*g* = -0.37) or panoramic ultrasound scanning (*g* = 0.38) methods were not showing even medium effect sizes on increasing biceps femoris FL (created via RevMan 5.4.1).

**Fig 13:**
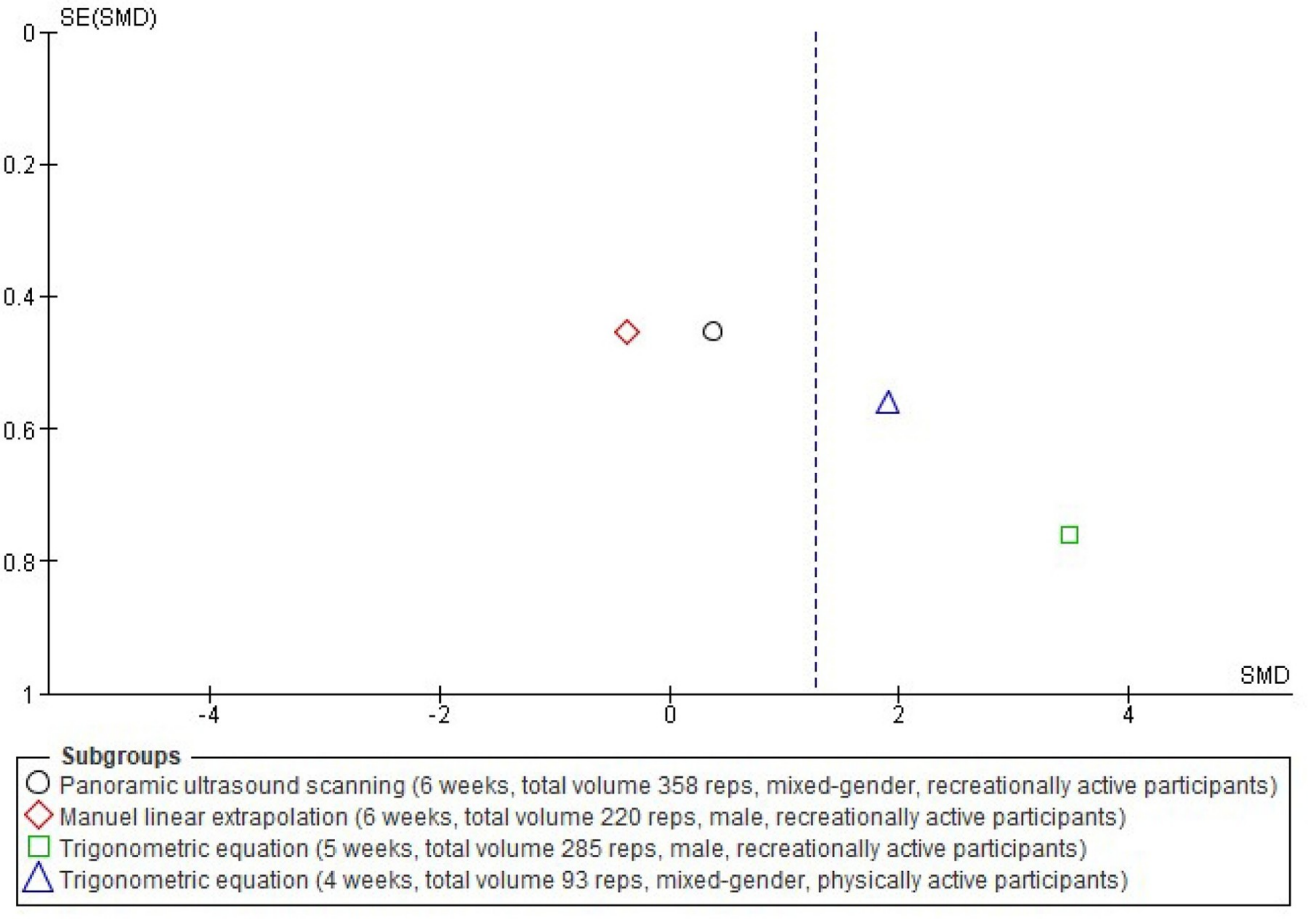
Funnel plot effects of 4–6 weeks of Nordic hamstring exercise on the biceps femoris FL based on ultrasound assessment and extrapolation methods. The red coloured square represents a study that used the manual linear extrapolation method, the black coloured circle represent a study that used the panoramic ultrasound scanning method, and the green coloured square and blue coloured triangle represent studies that used the trigonometric equation method. The asymmetry in the figure means a publication bias between the study groups that were used different ultrasound assessment methods (created via RevMan 5.4.1). Acronyms: SE(SMD), standard error of standardised mean differences; SMD, standardised mean difference.

Four studies [51, 56–58] with 4–6 weeks duration and with similar participants’ physical activity levels pooled in a meta-analysis in different subgroups based on the ultrasound assessment and extrapolation method for better understanding the possible effects of the total volume of the NHE and on the effect size estimation of the NHE on biceps femoris FL, As a difference, the mid-training data (5 weeks of NHE training and the control group) of Bourne et al. [51] employed this time in the meta-analysis for having closer total volumes between the studies. A forest plot in Fig 12 and a funnel plot in Fig 13 show the studies’ effect sizes. Despite the similar physical activity levels of the participants, four weeks [56] and five weeks [51] of NHE interventions used trigonometric equation methods for estimating the FL showed large effects sizes on increasing biceps femoris FL, while the six weeks of NHE interventions using the MLE [58] or panoramic ultrasound scanning [57] methods were not showing even medium effect sizes on increasing biceps femoris FL.

## Discussion

To the best of our knowledge, this was the first systematic review performing meta-analyses that compared the effects of eccentric exercise, including NHE, on biceps femoris FL between the RCTs based on an equation method, the MLE method and panoramic ultrasound scanning for estimating biceps femoris fascicle length. Among the previous meta-analyses, Cuthbert et al. [30] reported that NHE has a very large effect size (g ≥ 2.58) on increasing biceps femoris FL. Later, Medeiros, Marchiori and Baroni [31] estimated the effect size of the NHE as 0.97 ([-0.46, 1.48]). Additionally, Gérard et al. [29] found that eccentric strength training leads to a 1.97 cm ([1.48, 2.46] increment in biceps femoris FL. However, the findings of this meta-analysis differ from previous reviews. First, the effect sizes of the NHE were small (*g* = 0.23 [-1.02, 1.47], small (g = 0.38 [-0.50, 1.27]) and large (g = 1.98 [0.52, 3.44]) based on the MLE, panoramic ultrasound scanning and equation methods, respectively. Second, eccentric training leads 0.02 cm ([-0.13, 0.17], I^2^ = 55%), 0.47 cm ([0.15, 0.80], I^2^ = 0%), and 1.84 cm ([1.33, 2.34], I^2^ = 52%) increases in biceps femoris FL based on the MLE, panoramic ultrasound scanning and trigonometric equation methods, respectively. Additionally, eccentric training has a small effect based on the MLE method (*g* = 0.29 [-0.26, 0.85]), a medium effect based on the panoramic ultrasound assessments (*g* = 0.72 [0.17, 1.28])) and a large effect based on the trigonometric equation method (*g* = 2.20 [0.99, 3.41]).

Despite the fact that the equation method is validated by Kellis et al. [60] for estimating biceps femoris FL, Franchi et al. [33] have recently pointed out that the trigonometric equation method [60] overestimates 1.91 ± 2.1 cm biceps femoris FL compared to panoramic ultrasound (extended field of view) images. In contrast, the manual MLE method and panoramic ultrasound images had no significant differences between them [33]. In the case of this systematic review, three [51, 52, 56] of the eight RCTs used the trigonometric equation method [60]; three RCTs used the manual MLE method [54, 55, 58] and two RCTs employed panoramic ultrasound scanning [53, 57] for calculating the biceps femoris FL. Although initially large effect sizes for the eccentric training and NHE were found to increase biceps femoris FL without considering the calculation methods, subgroup analyses of this review detected differences between the ultrasound scanning and extrapolation methods. This systematic review detected large effect sizes only for those studies that applied trigonometric equation methods to estimate biceps femoris FL when considering the methods. The meta-analyses and subgroup analyses results showed that the eccentric strength training, including NHE, did not show any large effect on the size of biceps femoris FL based on the studies that applied the MLE method and panoramic ultrasound scanning. Additionally, a previous study found a poor agreement between ultrasound assessments using a trigonometric equation method for estimating biceps femoris FL and diffusion tensor MRI measurements on the biceps femoris FL [61]. However, more comparisons between the existing ultrasound and MRI measurement techniques are needed to having an overall idea about the agreement level between MRI and ultrasound assessments of biceps femoris FL. Furthermore, developing a gold standard measurement method, e.g. freehand three-dimensional ultrasound scanning, for biceps femoris FL measurements is needed, as stated by Franchi and colleagues [33].

There might be a possible underlying overestimation of the effect sizes reported by those studies that used the equation method for estimating the biceps femoris FL compared to the MLE and panoramic ultrasound scanning methods. However, this argument still needs evidence. Further studies might be conducted to compare the effects of eccentric training based on the ultrasound assessment and extrapolation methods. Additionally, the relevant scientific community could consider reaching a consensus for biceps femoris FL measurements to assess the impacts of training on this parameter by providing more comparable results between interventions.

In addition to these issues, missing standard deviations of the mean changes from baseline is critical when performing a meta-analysis of RCTs. A lower SD can produce a higher effect size or vice versa. The Cochrane handbook for Systematic Reviews of Interventions [32] describes missing SDs of the mean changes from baseline as a common feature in the literature, and the same handbook identifies the importance of obtaining the SDs. The formula for calculating the SD changes from baseline, and it is difficult to obtain this missing outcome, as explained in the ’data extraction, analysis and synthesis’ section of this systematic review. Previously, a survey reported that 68% of Cochrane reviewers who were aiming to run a meta-analysis for a continuous outcome faced the missing mean or SD value problems, and 85% of the reviewers finally asked the authors of the studies to share their missing outcome data, 76% of whom eventually did not pool the studies with missing outcome data [62]. This systematic review followed the recommendations of the Cochrane Handbook for Systematic Reviews of Interventions [32]. Among the eight RCTs [51–58], only two RCTs [54, 58] reported the required mean change and SDs of the mean changes from baseline. Among the remaining six RCTs, the required data could be calculated from the in-text information that exact P values and standard errors of only one RCT [55] via the Calculator of the RevMan program (RevMan 5.4.1) [55]. The required data of the remaining five studies [51–53, 56, 57] were obtained by contacting the corresponding authors of the studies. Starting from this point, the methodology of this systematic review for obtaining precise data differs from previous meta-analyses that investigated the effects of eccentric strength training [29] or NHE [30, 31] on biceps femoris FL.

Cuthbert and colleagues’ method [30] for meta-analysis differed from this systematic review and other relevant systematic reviews in methods to calculate the effect size of the NHE on biceps femoris FL. Nevertheless, the remaining two systematic reviews [29, 31] conducted the meta-analyses based on the mean changes and SDs of the mean changes from baseline for intervention and control groups, allowing a comparison of the results with those of this review [29, 31]. Four meta-analyses were carried out using the common studies among the present systematic review and recent systematic reviews [29, 31] for all cases of continuous data of MD (cm), 95% CI, fixed effect (FE); MD (cm), 95% CI, RE; SMD (effect size: Hedge’s (adjusted) g), 95% CI, FE; and SMD (effect size: Hedge’s (adjusted) g), 95% CI for establishing the proposed comparisons. All the results are shown in four funnel plots and four forest plots created by the RevMan computer program in S4 File. Additionally, Table 2 demonstrates the meta-analyses results based on the data of this review and the systematic reviews of Gérard et al. [29] and Medeiros, Marchiori& Baroni [31] for common studies. Based on the results, the reported data of previous systematic reviews [29, 31] produced results that were close to the actual centimetre changes in biceps femoris FL for common individual eligible studies [51, 55–57]. However, the reported data of both meta-analyses [29, 31] failed to precisely estimate actual effect sizes of the eccentric strength training or NHE on the biceps femoris FL due to miscalculations of the SDs of mean changes from the baseline. Therefore, this strongly suggests that future meta-analyses for continuous outcomes of RCTs related to the effects of eccentric exercise interventions on the biceps femoris FL should follow the recommendations of the Cochrane Handbooks for Systematic Reviews of Interventions [32], which includes contacting the corresponding authors of eligible studies to obtain mean changes and SDs of the mean changes from the baseline for precise results. Conversely, one limitation of the present review might be the small number of eligible studies pooled in meta-analyses. Nevertheless, this systematic review included eight studies in the quantitative syntheses, more than the previous systematic reviews that included five [29, 30] or four [31] studies. Additionally, a further confounder in the analysis of this review is the heterogeneity of training interventions, which adds non-accountable variability to the outcomes measures.

**Table 2:** Comparisons of effect sizes and mean changes of each study based on the given in-text data between meta-analyses investigating effects of eccentric strength training on biceps femoris fascicle length.

Study												
	MD (cm), FE, 95% CI			MD (cm), RE, 95% CI			SMD (Effect size: Hedge’s (adjusted) g), FE, 95% CI			SMD (Effect size: Hedge’s (adjusted) g), RE, 95% CI		
	Present SR	Gérard et al. [29]	Medeiros, Marchiori& Baroni [31]	Present SR	Gérard et al. [29]	Medeiros, Marchiori& Baroni [31]	Present SR	Gérard et al. [29]	Medeiros, Marchiori& Baroni [31]	Present SR	Gérard et al. [29]	Medeiros, Marchiori& Baroni [31]
**Bourne et al. [51] (NHE vs control)**	**2.41** [1.84, 2.97]	**2.47** [2.36, 2.58]	**2.40** [1.66, 3.14]	**2.41** [1.84, 2.97]	**2.47** [2.36, 2.58]	**2.40** [1.66, 3.14]	**3.56** [2.05, 5.08]	**18.79** [12.23, 25.35]	**2.73** [1.44, 4.02]	**3.56** [2.05, 5.08]	**18.79** [12.23, 25.35]	**2.73** [1.44, 4.02]
**Bourne et al. [51] (HE vs control**)	**1.52** [1.08, 1.95]	**1.58** [1.47, 1.69]	NA	**1.52** [1.08, 1.95]	**1.58** [1.47, 1.69]	NA	**2.92** [1.58, 4.26]	**11.61** [7.50, 15.71]	NA	**2.92** [1.58, 4.26]	**11.61** [7.50, 15.71]	NA
**Mendiguchia et al. [54]**	**0.76** [-0.08, 1.60]	NA	**0.76** [-0.27, 1.79]	**0.76** [-0.08, 1.60]	NA	**0.76** [-0.27, 1.79]	**0.90** [-0.18, 1.99]	NA	**0.71** [-0.35, 1.77]	**0.90** [-0.18, 1.99]	NA	**0.71** [-0.35, 1.77]
**Potier et al. [55]**	**1.03** [-0.18, 2.24]	**1.03** [0.91, 1.15]	NA	**1.03** [-0.18, 2.24]	**1.03** [0.91, 1.15]	NA	**0.69** [-0.18, 1.55]	**6.83** [4.45, 9.21]	NA	**0.69** [-0.18, 1.55]	**6.83** [4.45, 9.21]	NA
**Riberio-Alvares et al. [56]**	**1.61** [0.90, 2.32]	**1.63** [1.07, 2.19]	**1.63** [0.37, 2.89]	**1.61** [0.90, 2.32]	**1.63** [1.07, 2.19]	**1.63** [0.37, 2.89]	**1.89** [0.80, 2.99]	**2.44** [1.22, 3.66]	**1.09** [0.13, 2.04]	**1.89** [0.80, 2.99]	**2.44** [1.22, 3.66]	**1.09** [0.13, 2.04]
**Seymore et al. [57]**	**0.29** [-0.35, 0.93]	**0.29** [-0.18, 0.76]	**0.29** [-0.80, 1.38]	**0.29** [-0.35, 0.93]	**0.29** [-0.18, 0.76]	**0.29** [-0.80, 1.38]	**0.38** [-0.50, 1.27]	**0.51** [-0.38, 1.41]	**0.22** [-0.66, 1.10]	**0.38** [-0.50, 1.27]	**0.51** [-0.38, 1.41]	**0.22** [-0.66, 1.10]

Abbreviations: CG, Control Group, EG, Exercise Group, FE: Fixed Effect Model, MD, Mean Difference, NA, Not Applicable, RCT, Randomised Controlled Trial, RE: Random Effect Model, SMD, Standardised Mean Difference, SR, systematic review.

## Conclusions

Based on the meta-analyses and subgroup analyses of this systematic review, effect sizes on the eccentric strength training vary from small to large among the MLE, panoramic ultrasound scanning, and trigonometric equation methods. The only large effect size was detected in the subgroup consisting of the studies that used the trigonometric equation method for estimating biceps femoris FL. Likewise; the effect size of the NHE was large in the subgroup of the studies that used the trigonometric equation method for estimating biceps femoris FL. A consensus on ultrasound scanning techniques and biceps femoris FL estimation might provide comparable results between the exercise interventions targeting biceps femoris FL. Additionally, a future study can be conducted to compare the effects of eccentric training, which includes the NHE, based on the ultrasound assessment and extrapolation methods.

## Supporting information

S1 File Database searches. (DOCX)

S2 File PRISMA 2020 checklist. (DOCX)

S3 File Level of evidence of the meta-analyses. (DOCX)

S4 File Comparisons. (DOCX)
