# Supplementary material for: Heterogeneous effects of eccentric training and nordic hamstring exercise on the biceps femoris fascicle length based on ultrasound assessment and extrapolation methods: A systematic review of randomised controlled trials with meta-analyses
Source: PLoS One. 2021 Nov 9;16(11):e0259821. doi: 10.1371/journal.pone.0259821 (PMC8577763; doi:10.1371/journal.pone.0259821)

**Supporting Information S1 File. Database searches.**

Figure A. CINAHL Plus with full-text.


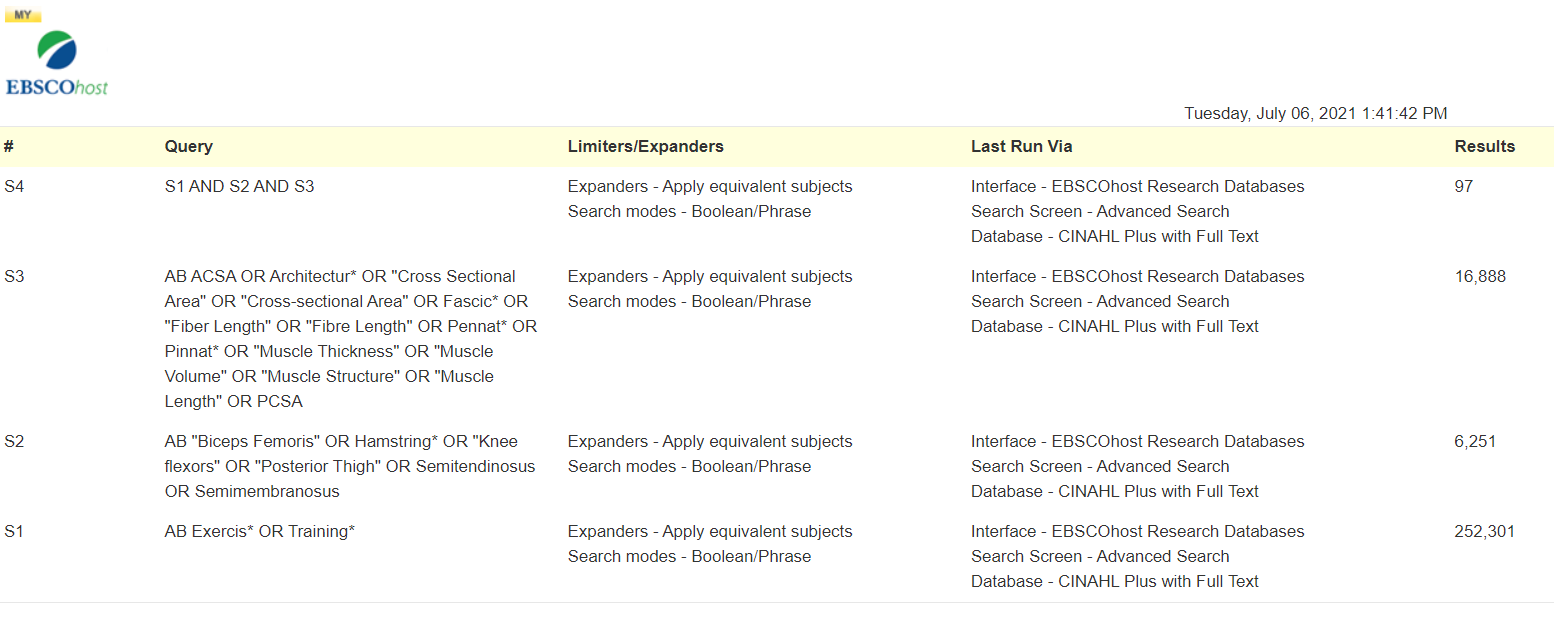


Table A. The Cochrane Central Register of Controlled Trials (CENTRAL).

Search Name:

Date Run: 06/07/2021 13:44:44

Comment:

ID Search Hits

#1 MeSH descriptor: [Exercise] explode all trees 25883

#2 Exercis* OR Training* 177668

#3 "Biceps Femoris" OR Hamstring* OR "Knee Flexors" OR "Posterior Thigh" OR Semitendinosus OR Semimembranosus 3185

#4 ACSA OR Architectur* OR "Cross Sectional Area" OR "Cross-sectional Area" OR Fascic* OR "Fiber Length" OR "Fibre Length" OR Pennat* OR Pinnat* OR "Muscle Thickness" OR "Muscle Volume" OR "Muscle Structure" OR "Muscle Length" OR PCSA 6018

#5 #1 AND #2 24238

#6 #3 AND #4 AND 5 in Trials 94

Table B. PubMed.

| Search number | Query | Sort By | Filters | Results | Time |
| --- | --- | --- | --- | --- | --- |
| 6 | #3 AND #4 AND #5 | Most Recent | | 229 | 09:03:44 |
| 5 | #1 OR #2 | Most Recent | | 813,191 | 09:02:35 |
| 4 | ACSA OR Architectur* OR "Cross Sectional Area"[Title/Abstract] OR "Cross-sectional Area"[Title/Abstract] OR Fascic* OR "Fiber Length"[Title/Abstract] OR "Fibre Length"[Title/Abstract] OR Pennat* OR Pinnat* OR "Muscle Thickness"[Title/Abstract] OR "Muscle Volume"[Title/Abstract] OR "Muscle Structure"[Title/Abstract] OR "Muscle Length"[Title/Abstract] OR PCSA | Most Recent | | 243,366 | 09:01:44 |
| 3 | "Biceps Femoris"[Title/Abstract] OR Hamstring*[Title/Abstract] OR "Knee flexors"[Title/Abstract] OR "Posterior Thigh"[Title/Abstract] OR Semitendinosus[Title/Abstract] OR Semimembranosus[Title/Abstract] | Most Recent | | 16,555 | 08:59:55 |
| 2 | Exercis*[Title/Abstract] OR Training*[Title/Abstract] | Most Recent | | 716,114 | 08:51:32 |
| 1 | "Exercise"[Mesh] | Most Recent | | 212,935 | 08:50:22 |
|  |  |  | |  |  |

Figure B. OpenGrey.


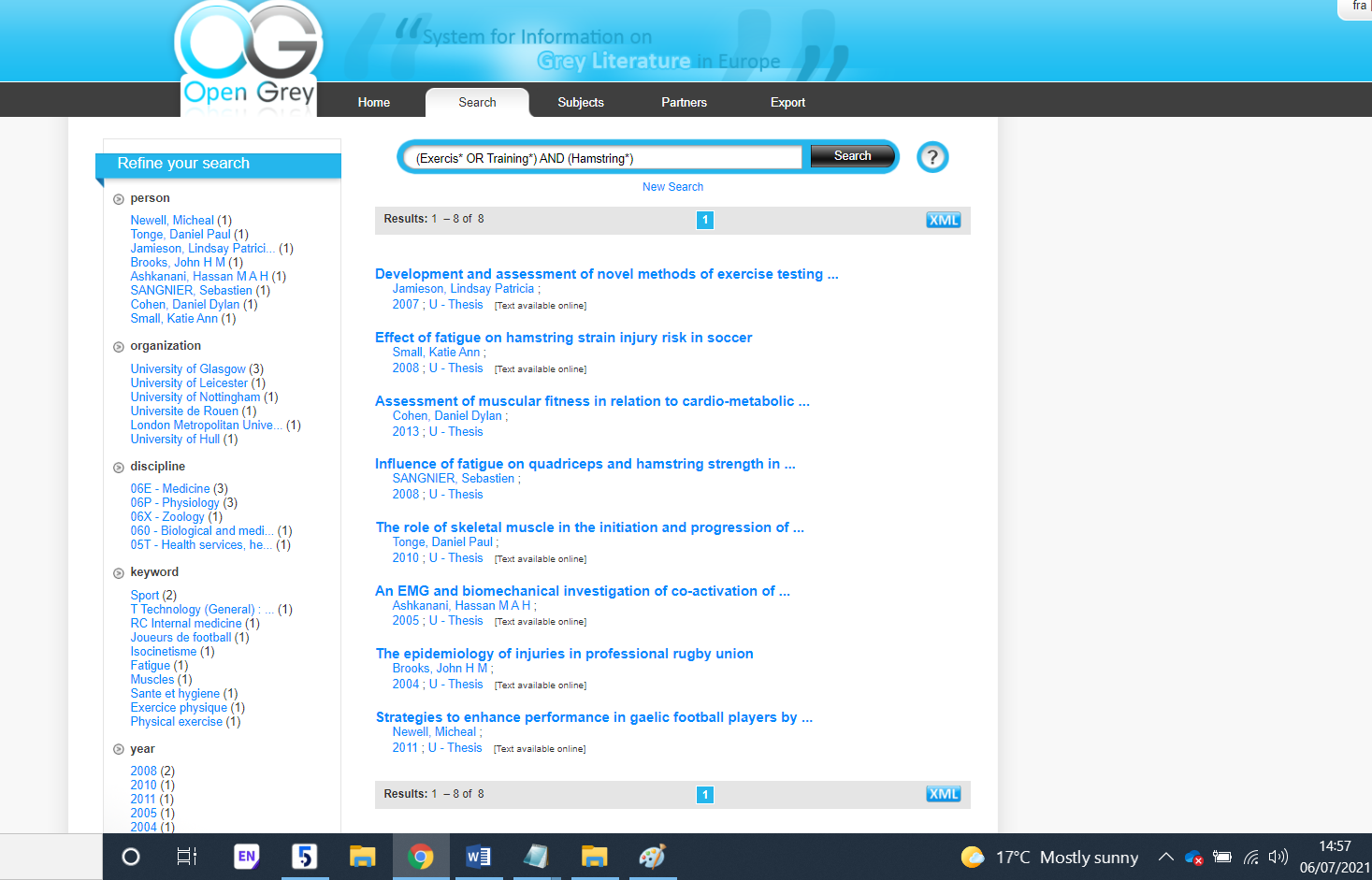

Supplement: S1 File — (DOCX) [file pone.0259821.s001.docx]
