# Supplementary material for: Heterogeneous effects of eccentric training and nordic hamstring exercise on the biceps femoris fascicle length based on ultrasound assessment and extrapolation methods: A systematic review of randomised controlled trials with meta-analyses
Source: PLoS One. 2021 Nov 9;16(11):e0259821. doi: 10.1371/journal.pone.0259821 (PMC8577763; doi:10.1371/journal.pone.0259821)
Supplement: S3 File — (DOCX) [file pone.0259821.s003.docx]

**Supporting Information S3 File. Level of evidence of the meta-analyses.**

Table A. Eccentric training (created via GRADEpro GDT).

| **Certainty assessment** | | | | | | | **№ of patients** | | **Effect** | | **Certainty** | **Importance** |
| --- | --- | --- | --- | --- | --- | --- | --- | --- | --- | --- | --- | --- |
| **№ of studies** | **Study design** | **Risk of bias** | **Inconsistency** | **Indirectness** | **Imprecision** | **Other considerations** | **Effects of eccentric training on the biceps femoris fascicle length based on the ultrasound extrapolation methods** | **placebo** | **Relative (95% CI)** | **Absolute (95% CI)** |  |  |
| **Effects of eccentric training on the biceps femoris fascicle length based on the ultrasound extrapolation methods** | | | | | | | | | | | | |
| 8 | randomised trials | serious ^a^ | very serious ^b^ | not serious | serious ^c^ | publication bias strongly suspected very strong association ^d^ | 106 | 105 | - | SMD **1.06 higher** (0.44 higher to 1.68 higher) | ⨁◯◯◯ VERY LOW |  |
| **Effects of eccentric training on the biceps femoris fascicle length based on the ultrasound extrapolation methods - Panoramic ultrasound scanning** | | | | | | | | | | | | |
| 2 | randomised trials | serious ^e^ | not serious | not serious | serious ^c^ | none | 28 | 26 | - | SMD **0.72 higher** (0.17 higher to 1.28 higher) | ⨁⨁◯◯ LOW |  |
| **Effects of eccentric training on the biceps femoris fascicle length based on the ultrasound extrapolation methods - Manual linear extrapolation** | | | | | | | | | | | | |
| 3 | randomised trials | serious ^f^ | not serious | not serious | serious ^c^ | none | 38 | 39 | - | SMD **0.29 higher** (0.26 lower to 0.85 higher) | ⨁⨁◯◯ LOW |  |
| **Effects of eccentric training on the biceps femoris fascicle length based on the ultrasound extrapolation methods - Trigonometric equation** | | | | | | | | | | | | |
| 3 | randomised trials | serious ^g^ | very serious ^b^ | not serious | serious ^c^ | publication bias strongly suspected very strong association ^h^ | 40 | 40 | - | SMD **2.2 higher** (0.99 higher to 3.41 higher) | ⨁◯◯◯ VERY LOW |  |

**CI:** Confidence interval; **SMD:** Standardised mean difference

#### Explanations

a. None of the 8 RCTs mentioned allocation concealment. Only one study performed participant blinded assessment. Three studies did not perform an assessor-blinded intervention. Three studies did not perform a reliability study.

b. I2 ≥ 75%

c. Large CI, low sample size

d. Detected in the funnel plot

e. No concealment, no blinded participants, no assessor-blinded in a study

f. No concealment in 3 studies, no reliability in one study, no blinded participants, no assessor-blinded in one study, no reliability in one study.

g. No concealment in 3 studies. No participants were blinded in 2 studies, no assessor-blinded in 1 study, no reliability was performed in 2 studies.

h. Possible overestimation was seen in funnel plots.

Table B. Nordic hamstring exercise (created via GRADEpro GDT).

| **Certainty assessment** | | | | | | | **№ of patients** | | **Effect** | | **Certainty** | **Importance** |
| --- | --- | --- | --- | --- | --- | --- | --- | --- | --- | --- | --- | --- |
| **№ of studies** | **Study design** | **Risk of bias** | **Inconsistency** | **Indirectness** | **Imprecision** | **Other considerations** | **Effects of Nordic hamstring exercise on the biceps femoris fascicle length based on the ultrasound extrapolation methods** | **placebo** | **Relative (95% CI)** | **Absolute (95% CI)** |  |  |
| **Effects of Nordic hamstring exercise on the biceps femoris fascicle length based on the ultrasound extrapolation methods** | | | | | | | | | | | | |
| 6 | randomised trials | serious ^a^ | very serious ^b^ | not serious | serious ^c^ | publication bias strongly suspected strong association ^d^ | 57 | 58 | - | SMD **1.09 higher** (0.16 higher to 2.01 higher) | ⨁◯◯◯ VERY LOW |  |
| **Effects of Nordic hamstring exercise on the biceps femoris fascicle length based on the ultrasound extrapolation methods - Panoramic ultrasound scanning** | | | | | | | | | | | | |
| 1 | randomised trials | serious ^e^ | not serious | not serious | serious ^c^ | none | 10 | 10 | - | SMD **0.38 higher** (0.5 lower to 1.27 higher) | ⨁⨁◯◯ LOW |  |
| **Effects of Nordic hamstring exercise on the biceps femoris fascicle length based on the ultrasound extrapolation methods - Manual linear extrapolation** | | | | | | | | | | | | |
| 2 | randomised trials | serious ^f^ | serious ^g^ | not serious | serious ^c^ | none | 17 | 18 | - | SMD **0.23 higher** (1.02 lower to 1.47 higher) | ⨁◯◯◯ VERY LOW |  |
| **Effects of Nordic hamstring exercise on the biceps femoris fascicle length based on the ultrasound extrapolation methods - Trigonometric equation** | | | | | | | | | | | | |
| 3 | randomised trials | serious ^h^ | very serious ^b^ | not serious | serious ^c^ | publication bias strongly suspected very strong association ^i^ | 30 | 30 | - | SMD **1.98 higher** (0.52 higher to 3.44 higher) | ⨁◯◯◯ VERY LOW |  |

**CI:** Confidence interval; **SMD:** Standardised mean difference

#### Explanations

a. No allocation concealment, no blinded participants in 5 studies, no reliability performed for three studies, no assessor-blinded in 3 studies.

b. I2 ≥ 75%

c. Large CI, low sample size

d. Was seen in the funnel plot

e. No blinded assessor or participants,

f. No allocation concealment in 2 studies, no blinded participants in two studies, no reliability performed in one study.

g. I2 ≥ 50%

h. No allocation concealment in 3 studies, no participants blinded in 2 studies, no assessors blinded in 2 studies, no reliability performed for one study

i. Possible overestimations were seen in the funnel plot
