# Supplementary material for: Heterogeneous effects of eccentric training and nordic hamstring exercise on the biceps femoris fascicle length based on ultrasound assessment and extrapolation methods: A systematic review of randomised controlled trials with meta-analyses
Source: PLoS One. 2021 Nov 9;16(11):e0259821. doi: 10.1371/journal.pone.0259821 (PMC8577763; doi:10.1371/journal.pone.0259821)
Supplement: S4 File — (DOCX) [file pone.0259821.s004.docx]

**Supporting Information S4 File. Comparisons.**

**Comparisons between this meta-analysis and previous meta-analyses^1 2^**

Figure A. Funnel plot for mean difference (cm), 95% confidence interval, fixed effect.


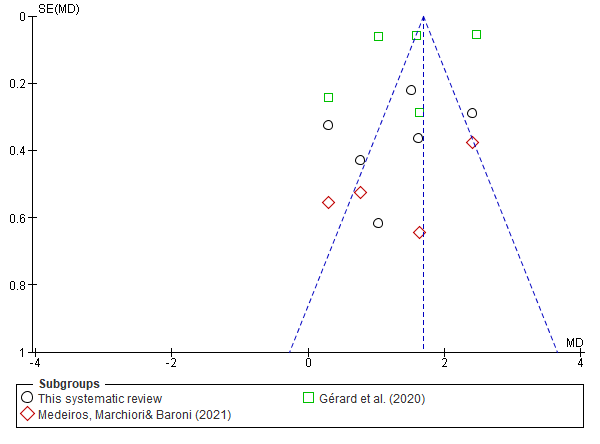


Figure B. Forest plot for mean difference (cm), 95% confidence interval, fixed effect.


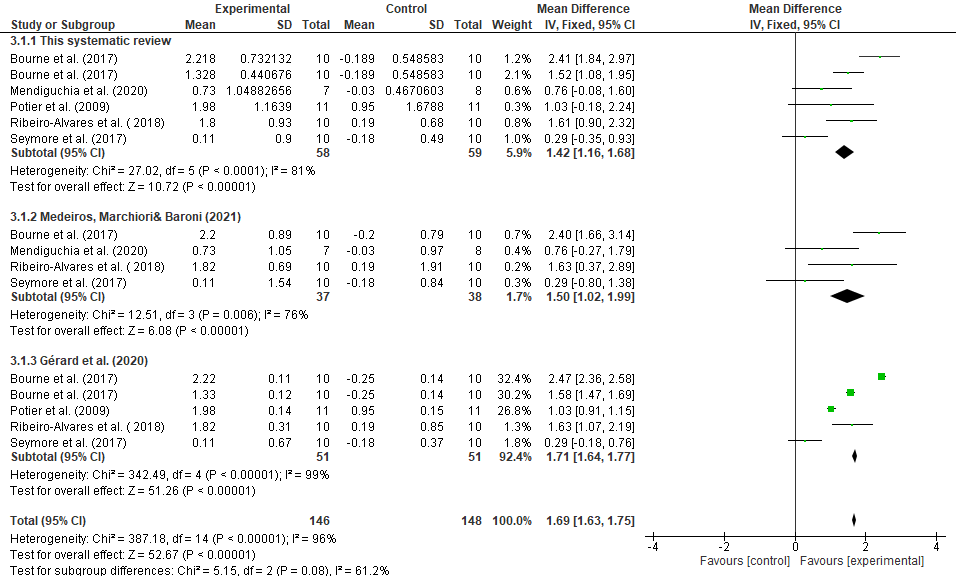


Figure C. Funnel plot for mean difference (cm), 95% confidence interval, random effect.


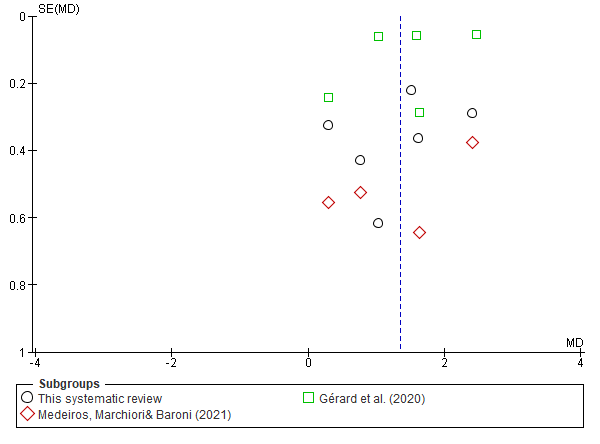


Figure D. Forest plot for mean difference (cm), 95% confidence interval, random effect.


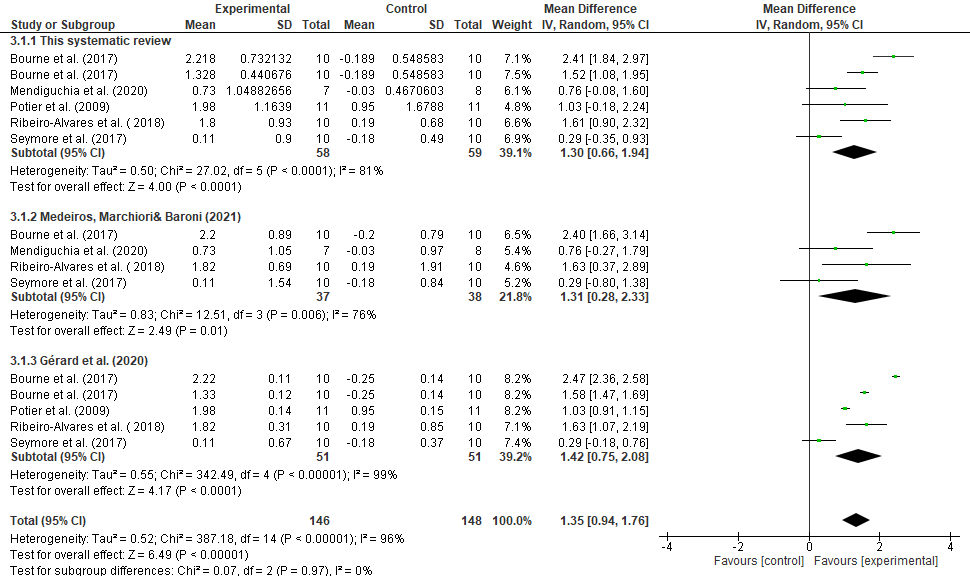


Figure E. Funnel plot for standardised mean difference (effect size (Hedge's (adjusted) g)), 95% confidence interval, fixed effect.


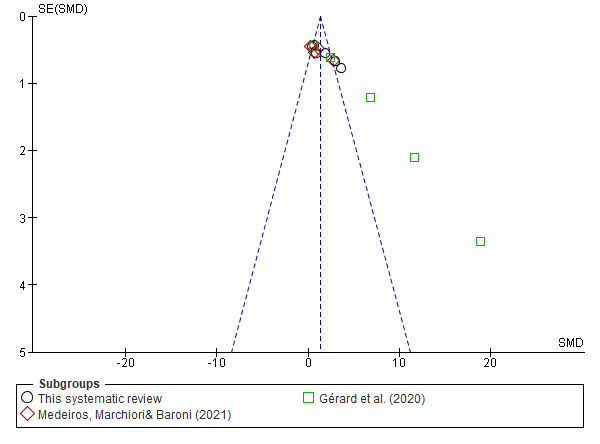


Figure F. Forest plot for standardised mean difference (effect size (Hedge's (adjusted) g)), 95% confidence interval, fixed effect.


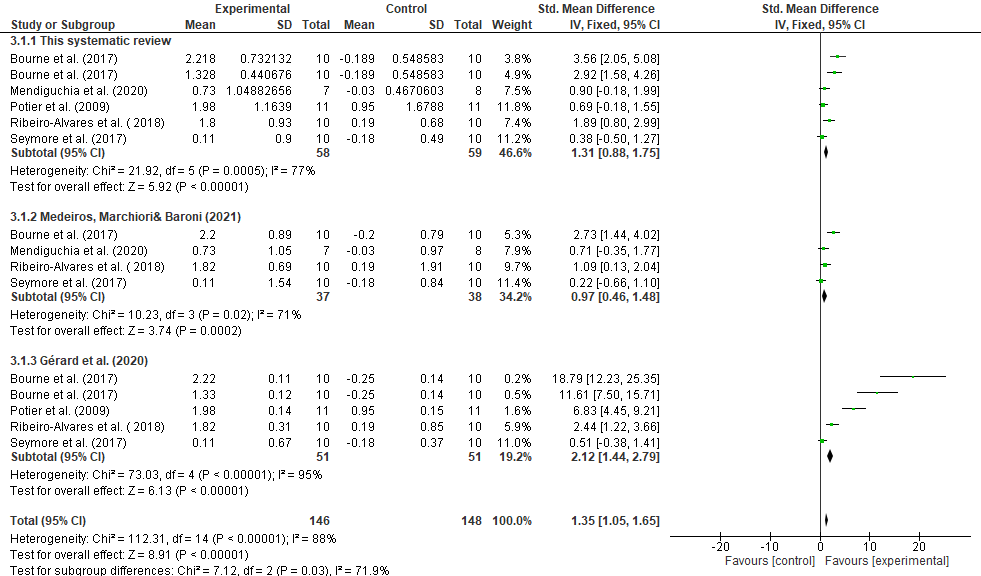


Figure G. Funnel plot for standardised mean difference (effect size (Hedge's (adjusted) g)), 95% confidence interval, random effect.


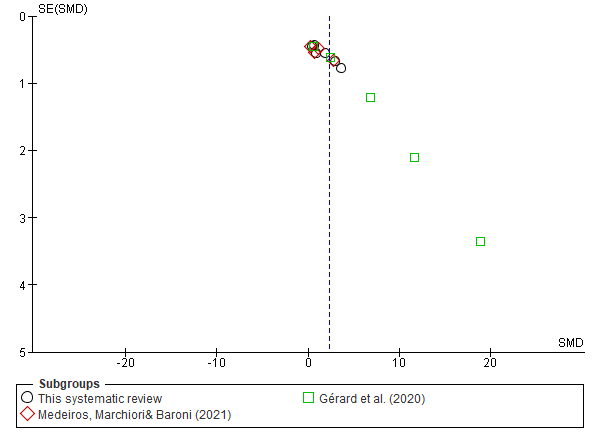


Figure H. Forest plot for standardised mean difference (effect size (Hedge's (adjusted) g)), 95% confidence interval, random effect.


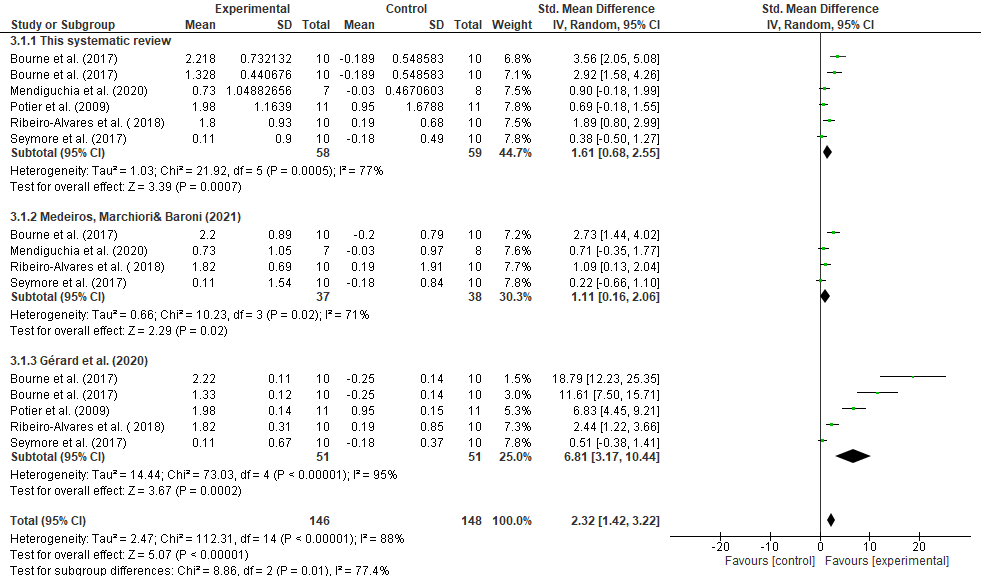


**References**

1. Gérard R, Gojon L, Decleve P, et al. The Effects of Eccentric Training on Biceps Femoris Architecture and Strength: A Systematic Review With Meta-Analysis. *J Athl Train* 2020;55(5):501-14. doi: 10.4085/1062-6050-194-19 [published Online First: 2020/03/29]

2. Medeiros DM, Marchiori C, Baroni BM. Effect of Nordic Hamstring Exercise Training on Knee Flexors Eccentric Strength and Fascicle Length: A Systematic Review and Meta-Analysis. *J Sport Rehabil* 2020:1-10. doi: 10.1123/jsr.2019-0388 [published Online First: 2020/10/14]
